# Chromosome 7 gain and DNA hypermethylation at the *HOXA10* locus are associated with expression of a stem cell related HOX-signature in glioblastoma

**DOI:** 10.1186/s13059-015-0583-7

**Published:** 2015-01-27

**Authors:** Sebastian Kurscheid, Pierre Bady, Davide Sciuscio, Ivana Samarzija, Tal Shay, Irene Vassallo, Wim V Criekinge, Roy T Daniel, Martin J van den Bent, Christine Marosi, Michael Weller, Warren P Mason, Eytan Domany, Roger Stupp, Mauro Delorenzi, Monika E Hegi

**Affiliations:** Neurosurgery, Lausanne University Hospital, Lausanne, 1011 Switzerland; Neuroscience Research Center, Lausanne University Hospital, Lausanne, 1011 Switzerland; Bioinformatics Core Facility, Swiss Institute for Bioinformatics, Lausanne, 1005 Switzerland; Department of Education and Research, University of Lausanne, Lausanne, 1011 Switzerland; Ben-Gurion University of the Negev, Beersheba, Israel; Department of Mathematical Modelling, Statistics and Bioinformatics, Ghent University, Ghent, Belgium; Department of Neurology/Neurooncology, Erasmus MC Cancer Center, Rotterdam, The Netherlands; Department of Medicine, Medical University Vienna, Vienna, Austria; Department of Neurology, University of Tübingen, Tübingen, Germany; Department of Neurology, University Hospital Zurich, Zurich, Switzerland; Princess Margaret Hospital, University of Toronto, Toronto, Canada; Department of Physics of Complex Systems, Weizmann Institute of Science, Rehovot, Israel; Department of Oncology, University Hospital Zurich, Zurich, 8091 Switzerland; Ludwig Center for Cancer Research, University of Lausanne, Lausanne, 1011 Switzerland; Department of Oncology, University of Lausanne, Lausanne, 1011 Switzerland; Present address: The John Curtin School of Medical Research, The Australian National University, Canberra, ACT 2601 Australia

## Abstract

**Background:**

*HOX* genes are a family of developmental genes that are expressed neither in the developing forebrain nor in the normal brain. Aberrant expression of a HOX*-*gene dominated stem-cell signature in glioblastoma has been linked with increased resistance to chemo-radiotherapy and sustained proliferation of glioma initiating cells. Here we describe the epigenetic and genetic alterations and their interactions associated with the expression of this signature in glioblastoma.

**Results:**

We observe prominent hypermethylation of the *HOXA* locus 7p15.2 in glioblastoma in contrast to non-tumoral brain. Hypermethylation is associated with a gain of chromosome 7, a hallmark of glioblastoma, and may compensate for tumor-driven enhanced gene dosage as a rescue mechanism by preventing undue gene expression. We identify the CpG island of the *HOXA10* alternative promoter that appears to escape hypermethylation in the HOX-high glioblastoma. An additive effect of gene copy gain at 7p15.2 and DNA methylation at key regulatory CpGs in *HOXA10* is significantly associated with HOX-signature expression. Additionally, we show concordance between methylation status and presence of active or inactive chromatin marks in glioblastoma-derived spheres that are HOX-high or HOX-low, respectively.

**Conclusions:**

Based on these findings, we propose co-evolution and interaction between gene copy gain, associated with a gain of chromosome 7, and additional epigenetic alterations as key mechanisms triggering a coordinated, but inappropriate, *HOX* transcriptional program in glioblastoma.

**Electronic supplementary material:**

The online version of this article (doi:10.1186/s13059-015-0583-7) contains supplementary material, which is available to authorized users.

## Background

Glioblastoma (GBM) is an aggressive brain tumor with a median survival of only 15 months. Despite remarkable efforts targeting prominent pathogenetic biological features of GBM, efficacy of novel drugs has been disappointing and significant gains in overall survival have not been made since the introduction of combined radio-chemotherapy comprising TMZ [1]. GBM are notorious for their treatment resistance. This has been attributed to the deregulation of major tumor suppressing and oncogenic pathways [2], tumor heterogeneity [3], and exhibition of stem cell-like properties by so called tumor stem cells, or glioma initiating cells (GICs) [4]. GICs represent a subpopulation(s) of tumor cells and are believed to, first, give rise to tumor progeny due to their self-renewing capacities, and second, resist radio- and chemotherapy [5,6].

In line with the notion of GICs’ contribution to treatment resistance, we earlier reported a self-renewal-related, *HOX*-dominated gene expression signature in GBM associated with significantly worse outcome in patients homogenously treated in a clinical trial with combined chemo-radiotherapy comprising the alkylating agent temozolomide. This association was independent of the predictive effect of *MGMT* methylation or age [7]. The abnormal expression of a *HOX* gene signature has been confirmed recently in GICs, where it has been functionally associated with their glioma initiating potential [8]. The importance of *HOX* gene expression for gliomagenesis and treatment resistance to temozolomide has been emphasized in several studies [8-12]. In 2006, Krivtsov and colleagues first described the inappropriate expression of a *HOX* gene signature in acute myeloid leukemia (AML) [13]. The authors showed in an elegant experimental mouse model that acquisition of this stem cell related *HOX* gene signature was associated with MLL-A9 fusion gene-induced leukemogenesis from committed progenitors of the granulocyte lineage, demonstrating for the first time that acquisition of stem cell properties in committed progenitor cells can lead to tumorigenesis.

*HOX* genes are a highly conserved family of genes encoding homeodomain transcription factors that provide anterior and posterior axial coordinates to vertebrate embryos during development [14]. In mammals, there are four paralogous *HOX* gene clusters organized on different chromosomes (CHRs). These gene clusters represent loci with extremely high gene density. In humans they are located on CHR7 (*HOXA*), CHR17 (*HOXB*), CHR12 (*HOXC*), and CHR2 (*HOXD*). The spatial organization of *HOX* genes is reflected in a 5′-posterior to 3′-anterior expression along the embryonal axes, termed spatial colinearity. Hence, expression of *HOXA9-13* is predominantly found in sites of the extremities, while *HOXA1-2* expression has been confirmed in, for example, the hindbrain. Although *HOX* genes are involved in the development of the hindbrain, other non-*HOX* homeobox genes regulate the development of the mid- and forebrain [15]. The forebrain comprises the ventricular and the subventricular zone, which harbors neural stem cells even in the adult brain, and has been proposed as origin of gliomas in the adult. Although this remains debated, mouse models have provided functional support [16].

Given that *HOX* genes are neither implicated in the developmental program of the brain nor expressed in the region of the adult brain that is thought to give rise to gliomas, we speculate that the HOX-signature is acquired during gliomagenesis, contributing stem cell properties. However, the mechanisms underlying the observed aberrant activation of *HOX* genes in GBM remain elusive. It has been proposed that the PI3K-pathway may be an important upstream regulator of *HOXA9* expression that is part of the HOX-signature [10]. A more recent report considered the involvement of MLL (*KMT2A*) in at least a subset of HOX-high expressing GBM. However, given the limited correlation reported, additional driver mechanisms triggering inappropriate *HOX* gene expression need to be considered [8]. Previous works have described a remarkable correlation of gene expression levels with gene dosage modulated by pathogenic copy number changes in cancer [17]. Most prominent among the *HOX* expression signature genes are *HOXA* genes, as corroborated by other labs [7,8,10]. The *HOXA* locus is located on CHR7 (7p15.2) that is affected by a copy number gain in up to 80% of GBM [18]. Most interestingly, gain of CHR7 has been proposed recently as the evolutionary first driver event in the development of primary GBM together with loss of one copy of CHR10 [19]. CHR7 harbors a number of potential driver genes, among many passengers that through CHR7 gain associated overexpression may drive/contribute to gliomagenesis. Of these, Ozawa *et al.* proposed PDGF as a driver gene for primary GBM, based on computational and experimental considerations. Previously we reported a low, but significant correlation between gene copy number of the *HOXA* cluster and expression of the HOX-signature [7]. However, only 42% of this patient cohort was found to be HOX-high. We therefore hypothesized that additional regulatory mechanisms are required to explain the abnormal expression of *HOXA* genes, with downstream effects on other HOX-signature genes. Here we present a model explaining the aberrant expression of the HOX-signature in GBM integrating multidimensional molecular data, comprising gene expression, gene copy number, and DNA methylation.

## Results

### Correlation between DNA methylation and gene expression in clinical GBM samples

**Figure 1:**
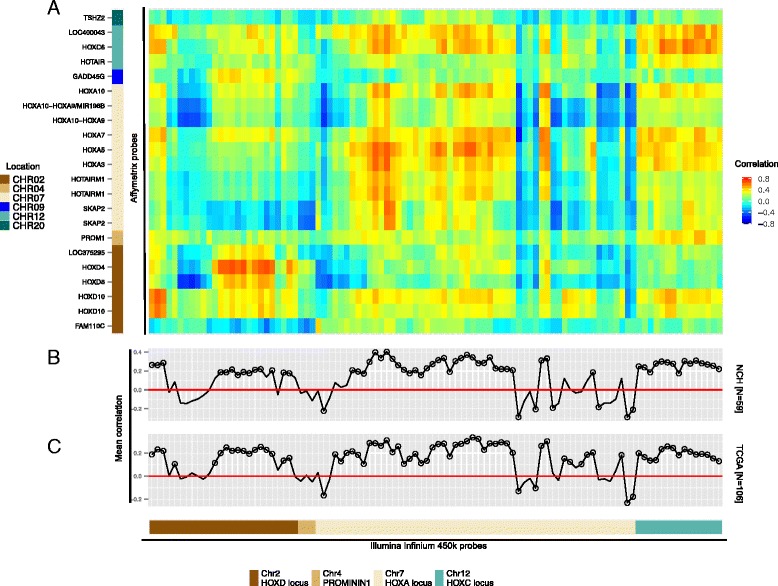
**Cross-correlation of HOX-signature gene expression and DNA methylation of top 100 associated CpGs. (A)** The heatmap visualizes the correlation between top 100 450k probes measuring DNA methylation of CpGs located in CpG islands of the *HOXD*, *PROMININ1*, *HOXA*, and *HOXC* loci (x-axis, see Additional file 1: Table S1 for detailed probe information) and the expression of 21 HOX-signature genes measured by 22 Affymetrix HG-133Plus2.0 probes (y-axis) in the NCH_EORTC GBM cohort. The probes on both axes are ordered according to chromosomal location. The chromosomal locations of the HOX-signature genes are color-coded. **(B)** Mean correlation between each selected CpG and expression of the HOX-signature genes of the NCH_EORTC dataset in A. The plot in **(C)** displays the mean correlation of the validation set comprising 106 GBM samples from TCGA using the same set of Infinium 450k probes and a total of 53 Agilent probes measuring expression of HOX-signature genes (see Additional file 1: Figure S3 for the corresponding heatmap). Circles highlight probes, which showed a statistically significant mean correlation between DNA methylation at measured CpG probes and the probes measuring expression of HOX-signature genes (q-values, FDR < =0.1). See also Additional file 2: Table S2 for list of Illumina Infinium 450k probes.

During gliomagenesis extensive epigenetic remodeling takes place, including global DNA hypomethylation and focused hypermethylation of promoter CpG-islands (CGIs) frequently silencing tumor suppressor genes. Recurrent gain of additional copies of CHR7 in GBM, often referred to as trisomy 7 in the literature, likely affects the *HOXA* locus that dominates the HOX-signature. The ‘HOX-signature’ refers to the signature identified previously [7], and comprises 21 genes covered by 22 probes of the HG-133Plus2.0 GeneChip (Additional file 1: Table S1). We hypothesized that epigenetic silencing mediated by methylation of CpGs in CGI-promoters of HOX-signature genes may compensate for tumor driven enhanced gene dosage as a rescue mechanism that could at least partially explain the differences in expression. In order to test this hypothesis we analyzed DNA methylation profiles of 59 GBM of the NCH_EORTC cohort obtained on the Illumina Infinium HumanMethylation450 BeadChip platform (450k). Indeed we found significant differences in the mean methylation across the *HOXA* locus on CHR7 (27,130,000 to 27,250,000; hg19 UCSC), when comparing GBM to non-tumoral brain (n = 4), with a generalized hypermethylation in the GBM samples as measured at 504 450k probes spanning the locus (*P* <0.001, two-sided t-test; Additional file 1: Figures S1 and S2). DNA methylation of the *HOXA* locus was significantly associated with gain of CHR7 (*P* <0.001, two-sided t-test), which may indicate compensation for increased gene dosage. In order to identify regulatory methylation patterns relevant for the expression of the whole HOX-signature the 400 probes annotated in CGIs of the 21 HOX-signature genes were subjected to principle component analysis. The top 100 CpG probes were selected based on their ranked cumulative contribution to the inertia of the DNA methylation table, hence representing the dominant contributors to the overall variability of the observed DNA methylation. The top 100 probes were predominantly located in the promoter CGIs of *HOXA* genes on CHR7, followed by *HOXD* genes on CHR2, *HOXC* genes on CHR12, and *PROMININ1* on CHR4 (Figure 1A and Additional file 2: Table S2, which contains mean correlation, *P* and q-values, and functional annotation for all 100 selected Infinium 450k probes for NCH_EORTC and TCGA data). The correlation between methylation of these CpGs and expression of each member of the HOX-signature was calculated. The heatmap of the correlation matrix (Figure 1A) visualizes the pattern of negative and positive correlation between DNA methylation and gene expression in the NCH_EORTC dataset. The strongest mean negative correlation (<= −0.28, (−0.28; 0.4)) between expression of HOX-signature genes and methylation was observed for a CpG probe located in the CGI associated with the alternative transcription start site of *HOXA10* (probe ID cg05092861 in UCSC CpG Island CHR7: 27219309–27219750, FDR <0.10) (Figure 1B, Additional file 2: Table S2). An adjacent CpG showed a similar correlation (cg01078824; −0.21, FDR <0.10; Additional file 2: Table S2).

### Determination of the correlation signature in an independent GBM dataset

Likewise we calculated the correlation matrix between DNA methylation and gene expression in an independent dataset of 106 GBM from The Cancer Genome Atlas (TCGA; Additional file 1: Table S4; mean correlation Figure 1C) visualized in a heatmap in Additional file 1: Figure S3. The similarity of the structure of the correlation matrices was remarkable for these two independent GBM datasets, with an RV-coefficient of 0.84 (simulated *P* value <0.001 (9,999 permutations), Additional file 1: Figures S3 and S4). Of note, while DNA methylation data was also generated on the 450k platform (used as the common dimension), the expression data originated from different platforms. In the GBM TCGA Agilent data the HOX-signature genes are covered with 53 probes (Additional file 1: Table S5), with probes missing for the ncRNA genes *MIR10B* and *HOTAIRM1*.

In order to evaluate the relevance of our findings in the context of the whole CHR7, we tested whether the apparent local enrichment of negative correlation between *HOXA* gene expression and DNA methylation at the *HOXA* locus was statistically significant. We determined the negative correlations between 711 RefSeq annotated CHR7 genes and their respective Illumina Infinium 450k probes and plotted the values according to genomic location of the genes (Additional file 1: Figure S5A). Gene set enrichment analysis (GSEA) revealed that the 10 *HOXA* genes were significantly enriched when testing their positions in the ranked list of the observed correlation coefficients of the 711 CHR7 genes as visualized in Additional file 1: Figure S5B (*P* value <0.001, Additional file 1: Table S3). A similar result was obtained for the TCGA dataset (*P* value <0.001; Additional file 1: Figure S5C; Table S3).

### The relationship DNA methylation/gene expression depends on CpG location

Next we were interested to evaluate the relationship of the mean correlation methylation/HOX-signature expression (Figure 1B and C) and the structural location of the respective CpGs using the Illumina annotation of the 450k probes (1st Exon, 3′UTR, 5′UTR, gene body, transcription start site (TSS) 1,500, TSS200). We observed that negative correlations between DNA methylation and the HOX-signature expression are primarily found for probes which are located either in the 1st exon of a gene, or within 200 bp of TSS, in line with canonical effects of promoter-CGI methylation on gene expression, while positive mean DNA methylation/gene expression correlations were found for probes located in the 3′UTR, 5′UTR, gene body and within 1,500 bp of TSS. This observation was consistent between the NCH_EORTC and the TCGA dataset (Additional file 1: Figure S6A and B, *P* value <0.01 and *P* value <0.05, one-way ANOVA).

### DNA methylation at *HOXA10* promoter CGI is lower in HOX-high than HOX-low GBM

The 59 GBM (NCH_EORTC) were classified into HOX-high (n = 25) or low (n = 34) based on iterative *k*-means clustering of the 22 Affymetrix probesets (Additional file 1: Figures S7 and S8). The average expression of the HOX-signature in the HOX-low group is not significantly different from respective measures in non-tumoral brain samples (*P* value = 0.9, all Welch’s Two-sample t-test) (Additional file 1: Figure S9A). In contrast, the higher mean expression levels of the HOX-signature in HOX-high samples are significantly different to both HOX-low (*P* value <0.01) and non-tumoral brain samples (*P* value <0.01). We observed significant differences in the degree of DNA methylation measured for probe cg05092861 between HOX-low and HOX-high samples, with a higher level of DNA methylation in HOX-low samples (*P* value <0.001). Both were different from non-tumoral brain (n = 4), which showed the lowest methylation levels (*P* value <0.001), although no expression is detected, while highest levels of DNA methylation were measured in HOX-low samples (non-tumoral brain < HOX-high < HOX-low, Additional file 1: Figure S9B). Similar differences were observed for the adjacent Infinium 450k probe cg01078824.

### Correlation between expression and gene copy number

**Figure 2:**
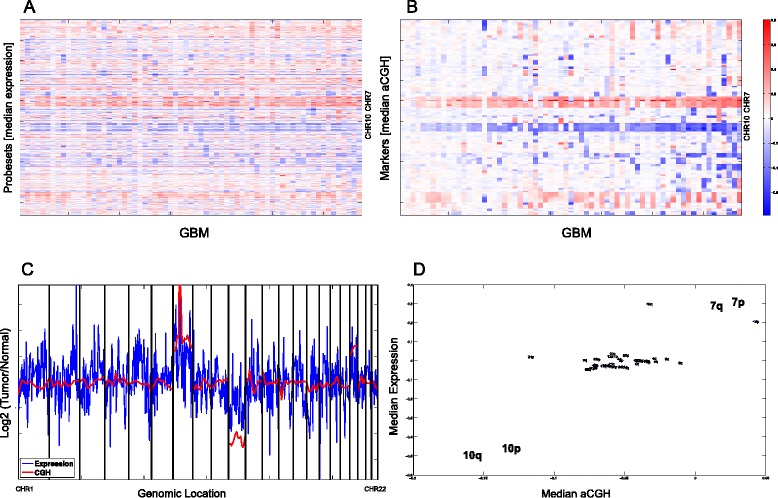
**Correlation of gene expression and gene dosage across the GBM genome.** Overall expression and aCGH in 64 GBM samples of the NCH-EORTC cohort are shown in fine resolution. **(A)** Gene expression is plotted relative to non-tumoral brain samples. Values were smoothened and interpolated. Every row is a probeset, and probesets are sorted by their genomic order. **(B)** aCGH data: Every row is a marker, and markers are sorted by their genomic order. In both **A** and **B**, the samples (columns) are sorted according to standard deviation in **B**. **(C)** For a single sample, the expression and aCGH values are shown. **(D)** For the same sample as in **(C)**, the median of all markers on every chromosome arm is plotted against the median relative expression of all probesets on the same chromosomal arm. Only chromosome arms for which there are probesets and aCGH markers are shown.

In order to investigate our hypothesis that gene dosage may contribute to the aberrant expression of the HOX-signature, we first tested the overall relationship between gene dosage and gene expression in our GBM dataset (NCH_EORTC). At the resolution of chromosomal arms that averages out regulatory factors affecting individual transcript levels, expression of the genes located on that arm and corresponding DNA copy number are strongly correlated (median Pearson correlation coefficient 0.73, standard deviation 0.18). The gene dosage effect on expression is particularly striking for gain of CHR7 and loss of CHR10, both hallmarks of GBM, as visualized in Figure 2 for the whole NCH-EORTC cohort (Figure 2A and B), or an individual GBM samples (Figure 2C and D).

### Gene dosage and DNA methylation impact HOX-signature expression

**Figure 3:**
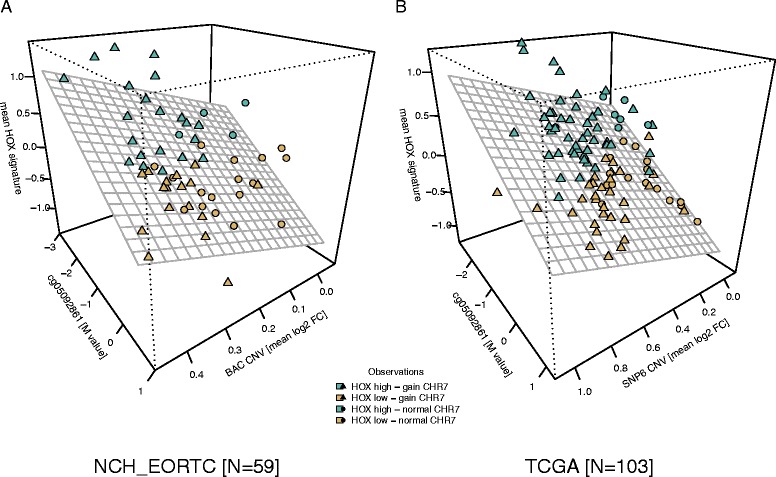
**HOX-signature expression levels are partially explained by DNA methylation and chr7p15.2 CNA.** The linear model in **(A)** visualizes the relationship between DNA methylation of the probe cg05092861 (M-values) and CNA at the cytoband chr7p15.2 (log2 fold-change (FC) over normal reference sample), and its association with mean HOX-signature expression levels of the 59 NCH_EORTC GBM samples. The projected plane represents the predicted response, and illustrates that mean HOX-signature levels are highest for samples, which have an increase in CNA and decrease in DNA methylation. As additional information, the copy number status calls (based on segmented information from BAC CGH data) of all samples are illustrated by circles (normal CHR7) and triangles (CHR7 gain). See Additional file 1: Figures S7 and S8 for the HOX signature based classification of samples into HOX-high and -low. Analogue to the model used for the NCH_EORTC data, **(B)** illustrates the linear model for the 103 TCGA GBM showing the combinatorial effect of CNA increase and DNA methylation decrease on the increase of mean HOX-signature expression levels. CHR7 copy number status of individual samples is illustrated by circles (normal) and triangles (gain). Additional file 1: Figure S11 illustrates the organization of TCGA samples into HOX-high and -low groups.

Next we tested the association between copy number alterations (CNA) at cytoband 7p15.2 that harbors the *HOXA* locus, methylation at the selected CpG (cg05098261) in the alternative promoter of *HOXA10*, and mean HOX-signature expression levels using an explanatory linear model. The M-values of the probe cg05092861 and the respective CNA values for cytoband 7p15.2 were introduced as independent variables, while the mean HOX-signature expression (based on scaled and centered gene expression) was the dependent variable. We observed a significant additive effect between CNA 7p15.2 and methylation at the selected probe in the alternative promoter of *HOXA10* as illustrated in Figure 3A. This model explains 32% of the variance of the HOX-signature expression levels (Table 1 - ‘Model 1’). The result supports the hypothesis that DNA methylation - at least in part - acts as an attenuating factor in samples with increased CNA at 7p15.2 in a manner that the effect of increased gene dosage on expression may be countered by the inhibitory effect of DNA methylation of *HOXA10* in GBM. Similar results were also observed in the TCGA subset of 103 GBM (for 3 of the 106 TCGA GBM the SNP6 CNA data were not available) as visualized in Figure 3B and summarized in Table 1. The model for the TCGA data explains 29% of the observed variance in HOX-signature expression (Table 1 - ‘Model 1’). Similar additive effects were observed for the adjacent CpG (cg01078824, Table 1 - ‘Model 2’).

**Table 1:** **Coefficients of linear models for HOX-signature expression in GBM datasets**

**Coefficients**	**Estimate**	**Std. Error** ^**a**^	**t value** ^**b**^	**Pr (>|t|)** ^**c**^	**R** ^**2d**^	**VIF** ^**e**^
NCH_EORTC (Model 1)					0.318	
(Intercept)	−0.793	0.166	−4.791	1.26e-05		
cg05092861	−0.453	0.110	−4.137	0.0001		1.001
CNA chr7p15.2 (BAC)	1.804	0.554	3.257	0.0019		1.001
NCH_EORTC (Model 2)					0.215	
(Intercept)	−0.204	0.168	−1.212	0.231		
cg01078824	−0.311	0.113	−2.754	0.008		1.002
CNA chr7p15.2 (BAC)	1.822	0.594	3.066	0.003		1.002
TCGA (Model 1)					0.289	
(Intercept)	−0.612	0.105	−5.822	7.07e-08		
cg05092861	−0.372	0.067	−5.542	2.44e-07		1.000
CNA chr7p15.2 (SNP6)	0.664	0.184	3.608	0.000		1.000
TCGA (Model 2)					0.254	
(Intercept)	−0.158	0.088	−1.785	0.077		
cg01078824	−0.356	0.071	−4.962	2.87e-06		1.023
CNA chr7p15.2 (SNP6)	0.799	0.191	4.186	6.13e-05		1.023

^a^Standard error.

^b^t-statistics.

^c^Two-sided *P* values of t-statistic.

^d^R-squared provides information about the variance explained by the model.

^e^Variance inflated factor (VIF), provides information about multi-collinearity of the model variables.

**Figure 4:**
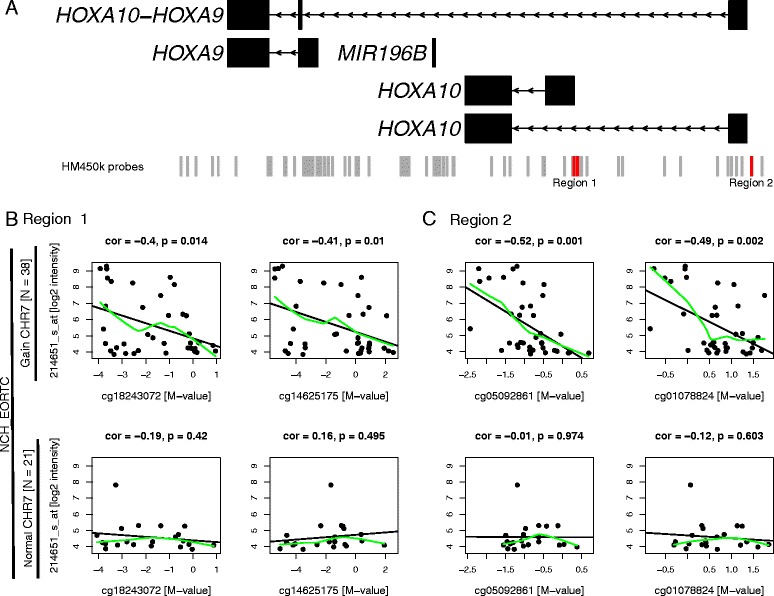
**Correlation of** ***HOXA10*** **expression and DNA methylation at the canonical and alternative promoter in 59 NCH_EORTC samples, stratified by CHR7 status.** The genomic organization of the *HOXA10* region on CHR7 is illustrated in **(A)** based on RefSeq annotation, retrieved from Ensembl using reference genome hg19/GRCh37. In addition the location of Infinium 450k probes are shown by grey bars at the bottom of the panel. Red bars highlight the location of the CpGs used for the plots in **(B)** and **(C)**. In **(B)** four X/Y plots show the correlation between DNA methylation of two CpGs located in the canonical *HOXA10* promoter (Region 1) and expression of the Affymetrix probe 214651_s_at. The top row of panels **(B)** and **(C)** show the expression and DNA methylation of samples with gain of CHR7 (n = 38). Bottom row of panels **(B)** and **(C)** show the corresponding data from samples with normal CHR7 status (n = 21). The CHR7 status was given by normal mixture model based on the weighted mean of the segmented copy number. Pearson’s product moment correlation coefficient (*cor)* and *P* values are shown above each plot. Black lines in the plots show the fit of linear regression, in green local regression using lowess smoothing is shown. Figure S10 in Additional file 1 shows the correlation between gene expression and DNA methylation for 103 TCGA samples.

Given the apparent central role of *HOXA10* in the HOX-signature, X/Y plots for both series of GBM, stratified by CHR7 status, illustrate the correlation between *HOXA10* expression alone (Affymetrix probe 214651_s_at) and methylation at the above identified 2 top CpGs (cg05092861, cg01078824) in the promoter of the putative non-coding alternative *HOXA10* transcript (NR_037939.1) and in addition at two CpGs (cg18243072, TSS200; cg14625175, exon1) located in the CGI of the promoter of the canonical *HOXA10* protein-coding transcript (NM_018951.3) (Figure 4B and C). In our dataset (NCH_EORTC) a significant negative correlation between expression and methylation was associated with CpG methylation in both regions for *HOXA10* expression in GBM with CHR7 gain (Spearman’s correlation *P* <0.05, Figure 4B and C). In contrast, no significant correlation was observed in GBM with normal CHR7 status (Figure 4B and C). Taken together, the findings for the HOX-signature gene *HOXA10* are in accordance with the model presented in Figure 3. Similar results were obtained for the TCGA dataset (Additional file 1: Figure S10B and C). The Spearman’s correlation coefficients for CpGs located in the two *HOXA10* promoters and *HOXA10* expression are available in Additional file 1: Table S6 for both datasets, stratified by CHR7 status. Additional file 1: Table S7 comprises the correlation coefficients for the top 100 CpGs and mean expression of the whole HOX-signature, stratified by CHR7 status for both NCH_EORTC and TCGA GBM.

### Transcriptome at the *HOXA* locus in GBM derived sphere (GS) lines

**Figure 5:**
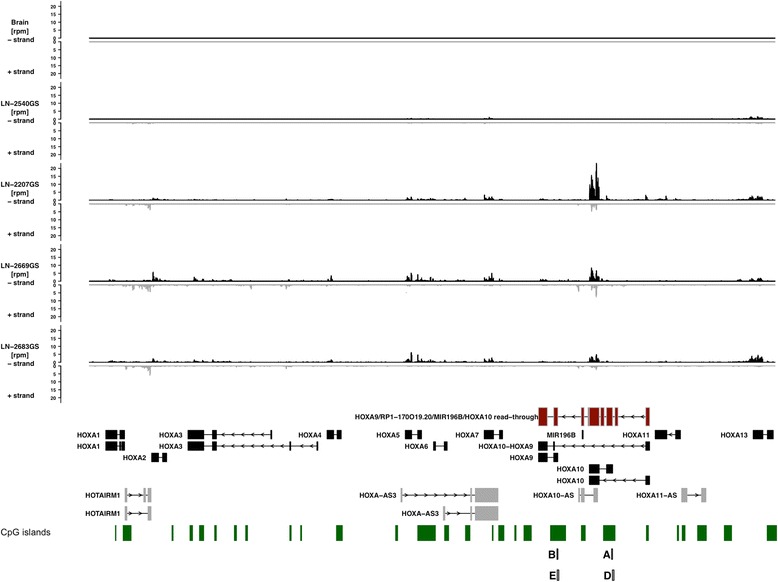
**RNA-Seq of four glioma sphere lines and one human brain sample.** Read densities at base pair resolution are shown across a 110 kB region of CHR7 (27,130,000-27,240,00) covering the human *HOXA* locus. Density of transcripts are presented separately for the minus (black) and plus (gray) strand and are shown as rpm/bp. The RefSeq annotations of the *HOXA* genes are shown as visual reference below the histograms. The location and structure of the putative HOXA9/10 read-through transcript is indicated in the RefSeq annotation track as a red gene model. See Additional file 1: Figure S12 for a Sashimi plot of RNA-Seq reads which support the presence of the read-through transcript. Locations of CpG islands (UCSC) are indicated by green bars. The gray boxes labelled A/B/D/E show the locations of amplicons used for ChIP-qPCR and methylation-specific clone sequencing (results shown in respective panels of Figure 6).

We sought to extend our findings to four GBM-derived sphere lines (GS lines) that retain GBM-relevant stem-cell properties and provide a better model of GBM than regular cell lines. Aligned read pile-ups derived from directed RNA-Seq data shown in Figure 5 illustrate the presence of both sense and anti-sense transcripts in the *HOXA* locus in three GS lines (LN-2207GS, LN-2669GS, LN-2683GS, thus designated as HOX-high models). In contrast, RNA-Seq reads generated from the HOX-low GS line LN-2540GS and human brain tissue (Ensembl Illumina Human BodyMap 2.0) show only very few and no aligned reads across the *HOXA* locus, respectively. These data confirm that the expression of transcripts from the *HOXA* locus in HOX-high GS lines is abnormal when compared to adult ‘normal’ brain. In the three HOX-high GS lines, the visualization of read alignments show that the majority of fragments originated from exons of *HOXA10*, with presence of some transcripts for *HOXA9* exons. We then used the RNA-Seq data to perform reference annotation based transcript assembly using cufflinks. This mapped transcript fragments of the three HOX-high GS lines to 12 of the 15 canonical HOXA sense and anti-sense transcripts. Additionally, using transcriptome re-assembly we found that the mapping of some of the transcript fragments suggests that a read-through transcript may be present, possibly consisting of exons of *HOXA10*, miR-196b, *HOXA9*, and the putative protein-coding RNA *RP1-170O19.20*. The presence of the AFFY-probes 214651_s_at and 209905_at in the HOX-signature that have been annotated as long non-coding RNA gene *HOXA10-HOXA9* lend some support to these findings. A model of this putative read-through transcript, based on the cufflinks reconstruction of the transcriptome of the GS lines, is visualized in the *HOXA* locus displayed in Figure 5. A Sashimi plot of the *HOXA10-9* locus (Additional file 1: Figure S12) illustrates the presence of transcript fragments, which span the exon/exon junctions of *HOXA9*, *HOXA10*, miR-196b, and RP1-170O19.20. However, further studies are warranted.

### Histone marks and promoter methylation at the *HOXA10/9* promoters

**Figure 6:**
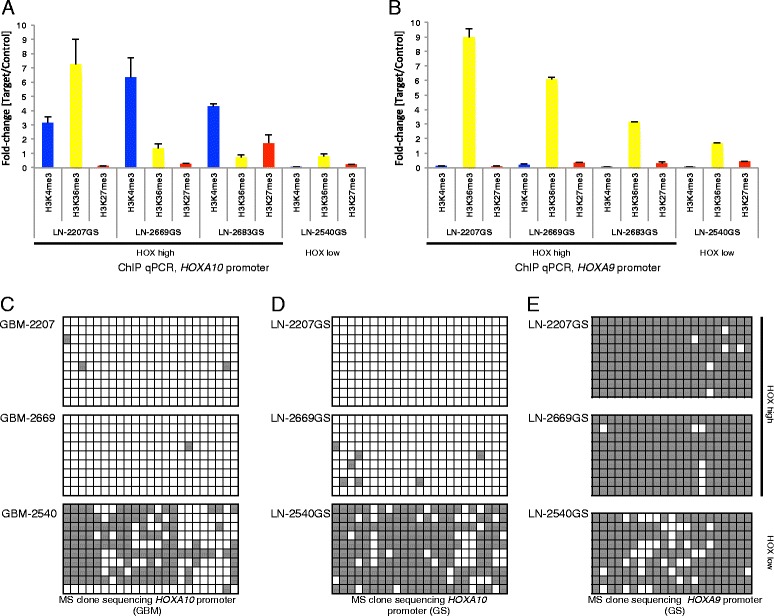
**Epigenetic features of the** ***HOXA10*** **and** ***HOXA9*** **promoters.** ChIP-qPCR is shown for three different histone marks, two associated with active transcription/open chromatin (H3K4me3, H3K36me3) and one indicative of transcriptional repression (H3K27me3), in our four glioma sphere lines at the *HOXA10* promoter **(A)** and the *HOXA9* promoter **(B)**. The measurements represent relative enrichment over IgG control (error bars represent SEM in duplicate experiments). Methylation-specific clone sequencing is shown for the CpGi located in the promoter of *HOXA10* in three primary GBM **(C)** and respective derived glioma sphere lines **(D)**. The *HOXA10* promoter of HOX-high GBM-2207 and GBM-2669, and the corresponding GS lines (LN-2207GS, LN-2669-GS) is unmethylated, in contrast to the HOX-low GBM-2540 and its corresponding GS line which exhibit both a highly methylated *HOXA10* promoter. **(E)** The CpGi located in the promoter of *HOXA9* is highly methylated in all three glioma spheres lines. Gray boxes represent methylated CpGs.

One important regulatory mechanism of transcription is the conformational state of chromatin, as mediated through various post-translational histone modifications. The observation that *HOXA* genes are actively transcribed in a subset of GBM and in three of our four GS lines indicates that the chromatin is in a permissive state. We tested this assumption by performing chromatin-immunoprecipitation (ChIP) in the four GS lines for three different histone marks: H3K4me3 - mark for active or poised promoters; H3K36me3 - indicative of transcriptional elongation; and H3K27me3 - a repressive mark, followed by qPCR at the promoters of *HOXA10* and *HOXA9* (Figure 6A and B). ChIP-qPCR revealed a predominant signal for H3K4me3 and presence of H3K36me3 in the HOX-high GS lines (LN-2207GS, LN-2669GS, LN-2683GS) in the *HOXA10* promoter, while they were absent in the HOX-low GS line LN-2540GS. In contrast, the predominant mark detected in the *HOXA9* promoter was H3K36me3, while the other marks were absent. In addition, we determined the methylation status using methylation-specific (MS) clone sequencing of the CpG island of the *HOXA10* promoter (CHR7:27212417–27214396) in three GS lines (2 HOX-high and 1 HOX-low) and their respective original GBM. The methylation pattern in the GS lines was highly similar to the original primary tumors from which they were isolated, excluding an *in vitro* artifact (Figure 6C and D). The *HOXA10* promoter was unmethylated in the two HOX-high GS lines LN-2207GS and LN-2669GS and their respective original GBM, and fully methylated in the HOX-low GS line LN-2540GS and the respective GBM-2540. This corroborates our finding of strong negative correlations between expression/methylation for 2 CpGs (cg18243072, cg14625175) interrogated in this region on the 450k BeadChip, as illustrated and annotated in X-Y-plots for both NCH_EORTC (Figure 4) and TCGA samples (Additional file 1: Figure S10). MS clone sequencing of the *HOXA9* promoter (CHR7:27205048–27205315) revealed full methylation also in the HOX-high GS lines (Figure 6E). Taken together, the marks for actively transcribed chromatin are in accordance with *HOXA10* expression. *HOXA10* is also translated into protein as we have shown previously for GS lines, including LN-2207GS and a series of GBM on a tissue micro array [7]. The observed enrichment for the elongation mark H3K36me3 at the fully methylated *HOXA9* promoter would be compatible with the presence of a putative read-through transcript of *HOXA10* and *HOXA9*, as suggested by our analyses of the RNA-Seq data (Figure 5 and Additional file 1: Figure S12) and the probe annotation from the HG-133Plus2.0 GeneChip.

### HOX-signature associated microRNAs

To test for the potential impact of regulatory microRNAs on the HOX-signature we analyzed the correlation between the mean expression of the HOX-signature in the TCGA Agilent data subset and the expression levels of individual microRNAs (Table 2). We found that the top correlated microRNAs (Spearman’s rho > = 0.5, *P* value <0.0001) were in fact transcribed from *HOX* loci (miR-196b - *HOXA*, miR-10a - *HOXB*, miR-10b - *HOXD*), known to have regulatory functions in *cis* [20]. However, the expression of these microRNAs was positively correlated, suggesting that the de-repression of *HOX* genes in GBM may lead to an overall aberrant co-expression of genes from the affected loci including the enclosed microRNAs. This is further supported by the observation that several long non-coding RNAs transcribed from the *HOXA* (*HOTAIRM1*) and *HOXC* loci (LOC400043, *HOTAIR*) and adjacent to the *HOXD* locus (LOC375295), were detected as part of the HOX-signature (Additional file 1: Table S1).

**Table 2:** **Correlation between microRNAs and mean HOX-signature expression in 106 GBM samples from TCGA, top 2 percentile positively and negatively correlated microRNAs**

	**Spearman’s rho**	***P*** **value (two-sided)**
**hsa-miR-196b (HOXA locus)**	0.69	<0.01
**hsa-miR-10b (HOXD locus)**	0.50	<0.01
**hsa-miR-196a (HOXB/C locus)**	0.5	<0.01
hsa-miR-148a	0.42	<0.01
hsa-miR-106b	0.35	<0.01
hsa-miR-25	0.32	<0.01
hsa-miR-496	0.31	<0.01
hcmv-miR-UL148D	0.3	<0.01
hsa-miR-337	0.29	<0.01
hsa-let-7c	0.29	<0.01
hsa-miR-130b	0.29	<0.01
hsa-miR-199a*	−0.27	<0.01
hsa-miR-223	−0.27	<0.01
hsa-miR-148b	−0.28	<0.01
hsa-let-7f	−0.29	<0.01
hsa-miR-125a	−0.29	<0.01
hsa-let-7d	−0.3	<0.01
hsa-miR-98	−0.3	<0.01
hsa-let-7 g	−0.33	<0.01
hsa-miR-143	−0.36	<0.01
hsa-miR-107	−0.37	<0.01
hsa-miR-145	−0.42	<0.01

Lines in **bold** highlight microRNAs transcribed from *HOX* loci.

### Relationship of the HOX-signature and molecular GBM subtypes

Next we sought to address how the HOX-signature was related to three established molecular GBM classification schemes: (1) the four GBM expression subtypes neural, proneural, mesenchymal, and classical as proposed by Verhaak and colleagues [21]; (2) the glioma CpG island methylator phenotype (G-CIMP) present in a subgroup of proneural GBM [22]; or (3) distinction of *MGMT* promoter methylated vs. unmethylated that has been shown to be highly predictive for benefit from alkylating agent chemotherapy [23]. Expression data from 473 GBM (TCGA, level 2 Agilent) were used to classify samples into either HOX-high (259) or low (214), based on k-means clustering (Additional file 1: Figure S13), and were annotated with the expression subtype classification, including G-CIMP, and *MGMT* promoter methylation status [2,24] (Additional file 1: Figure S14). We observed an enrichment of proneural GBM in the HOX-high group, while the proneural G-CIMP-positive GBM were under-represented (Additional file 1: Table S8, *P* value <0.001, Pearson’s Chi-squared test). No significant associations were found in the other three expression subtypes. No correlation was found with expression of PDGF that has been proposed as a gain of CHR7-associated driver gene for G-CIMP negative GBM [19]. Finally, we confirmed our previous finding from the NCH_EORTC dataset [7] that the *MGMT*-promoter methylation frequency was not different between the HOX-high and low groups (Additional file 1: Table S9, *P* value >0.35, Pearson’s Chi-squared test).

## Discussion

In the present study we sought to elucidate underlying molecular mechanisms triggering the inappropriate expression of a HOX-signature. Such a *HOX* gene dominated expression signature has been associated by others, and us with resistance to temozolomide and conferring of glioma-initiating properties [7,8,11,12].

The hypermethylation of the *HOXA* locus was associated with gain of CHR7, which is reminiscent of compensation for increased gene dosage, known from X-chromosome inactivation [25-28]. This is further supported by the observation that a significant correlation between DNA methylation and expression was only observed for samples with gain of CHR7 as visualized for *HOXA10*. The involvement of gene dosage mediated induction of HOX-signature expression is compatible with the observation that HOX-high GBM are under-represented in G-CIMP positive GBM, which reportedly have a much lower frequency of CHR7 gain [2]. The observed correlation between expression of the whole HOX-signature and DNA methylation suggested DNA methylation patterns permissive for expression. However, the regulatory effects leading to coordinated expression of *HOXA*, *C*, and *D* genes, and the other members of the signature, including the stem cell marker *PROM1* that are located on other chromosomes, are not yet explained. With the exception of the developing forebrain where *HOX* genes are repressed [29], the coordinated expression and silencing of *HOX* genes is well known from embryonic development. It involves changes of higher chromatin organization and complex regulation implicating long-range control mechanisms which are only partly understood [14].

*HOX* genes are tightly regulated through polycomb-repressor complex 2 (PRC2)-mediated tri-methylation of H3K27 [30]. Investigation of GS lines suggested loss of the repressive mark H3K27me3 and gain of the active mark H3K4me3 in the promoter of *HOXA10* protein-coding transcript variant 1 in HOX-high GS lines, and was associated with an unmethylated CGI. This observation is in accordance with the detection of HOXA10 protein in HOX-high GS lines, as well as in a subset of GBM [7]. In contrast, the HOX-low GS line lacked the active mark and displayed a methylated *HOXA10* promoter. Interestingly, the histone marks in the *HOXA9* promoter displayed enrichment of H3K36me3 in the HOX-high GS lines in conjunction with the fully methylated CGI suggested transcriptional elongation. This pattern of histone marks would also be compatible with presence of a *HOXA10/9* read-through transcript, as proposed by the RNA-Seq analysis and the respectively annotated HG-133Plus2.0 probes that are part of the HOX-signature. Little is known about the functional relevance of the putative long non-coding RNA gene *HOXA10-HOXA9.* It has been proposed as candidate for nonsense-mediated mRNA decay (NMD) [31-33].

Non-coding RNAs, like lincRNAs and microRNAs, can be involved in the regulation of *HOX* gene expression [34-36]. Our HOX-signature also includes several lincRNAs, which are transcribed from the different *HOX* loci: LOC400043 and *HOTAIR* from the *HOXC* locus, *HOTAIRM1* from the *HOXA* locus, and LOC375295 from the *HOXD* locus. For the lincRNAs *HOTAIR* and *HOTAIRM1* functions have been investigated. *HOTAIRM1* can regulate the expression of *HOXA* genes through facilitating conformational changes to the chromatin, in proximal distal manner [35-38]. An initial suspicion that *HOTAIRM1* and other ncRNAs could be directly involved in the regulation of the HOX-signature genes was tantalizing. However, their expression pattern, and the observation that the top three correlated microRNAs are actually transcribed from the *HOXA/B/C/D* loci, rather suggested that these small and long ncRNAs are more likely ‘caught in the storm’ of a coordinated, but inappropriate *HOX* transcriptional program.

## Conclusions

In conclusion, our results suggest that the aberrant expression of the HOX-signature, which confers stem-cell related properties and resistance to therapy, may be acquired through gene copy gain associated with CHR7 gain. Hypermethylation appears to compensate for gene copy gain at this locus in the HOX-low GBM, preventing CHR7 gain driven increase of expression, while in HOX-high GBM key CpGs in the *HOXA* locus escape hypermethylation. Gene copy gain and methylation at key CpGs in the promoter of *HOXA10* putative non-coding transcript variant 2 are strongly associated with the expression of the whole HOX-signature. These findings are remarkably reproducible in an independent GBM dataset from TCGA. The observed mechanism of escape from DNA hypermethylation may explain overexpression of other gliomagenesis relevant proto-oncogenes located on CHR7 and other loci affected by tumor-related increased gene dosage. Hence, further studies are warranted to investigate the co-evolution of gene copy number changes and epigenetic changes, including tumorigenesis-associated DNA methylation, to identify tumor relevant deregulated genes. Finally, the observation of compensatory DNA methylation at genes with potential proto-oncogenic function should be taken into account when considering epigenetic drugs.

## Methods

### GBM datasets and GBM derived sphere lines

Our patient cohort of 59 GBM patients (NCH_EORTC), for whom Affymetrix HG-133Plus2.0 gene expression and Illumina Infinium 450k DNA methylation data were available, has been treated within clinical trials [39,40]. Patients treated within EORTC 26981 had consented for translational research of their tumor tissues as part of the study protocol. All other patients gave informed consent according to the protocol approved by the local ethics committee (protocol F25/99) and the respective competent Swiss federal authorities (No 1.05.01.10-48). The study protocols conform to the World Medical Association Declaration of Helsinki [41]. Analysis of non-tumoral brain samples and the establishment of the GBM derived sphere lines (GS lines) LN-2207GS, LN-2540GS, LN-2669GS, and LN-2683GS, respective authentication, and the description of the respective original tumors have been published previously [7,42,43]. Briefly, GS lines were cultured under stem cell conditions using DMEM-F12 medium (Invitrogen, 10565–018) supplemented with human recombinant EGF and human recombinant basic FGF (Peprotech, AF-100-15 and 100-18B), 20 ng/mL each, and 2% B27 (Invitrogen, 17504); 50% of the medium was substituted twice weekly.

### Bisulfite treatment and methylation-specific (MS) clone sequencing, and DNA methylation profiling

DNA isolated from frozen tissues or cells was treated with bi-sulfite, and methylation profiling was performed using Infinium HumanMethylation450 BeadChip (Illumina). MS clone sequencing was performed as previously described [42]. See also Extended Experimental Procedures.

### RNA-Seq of glioma sphere transcriptomes and data analysis

Total RNA isolated from GS cells was depleted from ribosomal RNA and sequencing libraries were prepared using TruSeq Stranded Total RNA with Ribo-Zero Gold (Epicentre, Illumina), followed by paired-end sequencing on Illumina Hiseq (PE 2x50 bp; NXTGNT, University of Gent, Belgium). Details on read-alignment, transcriptome reconstruction and data visualization can be found in the Extended Experimental Procedures.

### Chromatin immunoprecipitation followed by quantitative PCR (ChIP-qPCR)

Chromatin was prepared using the MAGnify Chromatin Immunoprecipitation System (Invitrogen), precipitated with antibodies targeting the interrogated histone marks, and DNA quantified using qPCR as previously described [42]. See Extended Experimental Procedures for details on the procedure and antibodies used for immunoprecipitation.

### aCGH dataset

For the NCH_EORTC samples, the Bacterial Artificial Chromosome (BAC) aCGH data were acquired by UCSF Humarray 2.0 and 3.0 platforms containing 2,428 BACs, each spotted in triplicate, distributed over the human genome with an average resolution of 1.4 Mb [18,44]. Details on data processing and analysis are presented in the Extended Experimental Procedures.

### Selection of TCGA samples included in the analysis and data processing

We applied two criteria to select samples from TCGA for our validation dataset: First, the gene expression platform should have sufficient coverage of the HOX-signature in terms of probes measuring expression levels of all 21 genes. Second, DNA methylation should be measured with the Illumina Infinium 450k platform, as this provided us a common dimension necessary to assess the similarity between the two datasets. Details on the sample selection are presented in the Extended Experimental Procedures.

### Data analysis

Unless otherwise stated, all data processing, analysis, and visualization were performed in R version 3.1.0 [45]. Packages for specific data types and tasks are listed in the relevant sections.

### Processing and normalization of Illumina Infinium 450k DNA methylation data

The methylation array data of samples was loaded into R and processed using the BioConductor package ‘minfi’. The detection *P*-values, probabilities that the target sequence signal was distinguishable from the background, were used to exclude probes with poor quality. The probes that are unsuccessfully measured (p-detection >0.01) in more than 1% of samples were dropped from the dataset. The DNA methylation data from the 450k BeadChip were preprocessed as in Genome Studio (software provided by Illumina) and they were summarized by M-values as recommended by Du *et al.* [46].

### Processing and normalization of Affymetrix gene expression data

The expression intensities for all probe sets from Affymetrix CEL-files were estimated using robust multiarray average (RMA) with probe-level quantile normalization and the Normalized Unscaled Standard Errors values (NUSE) were used to assess the relative quality of arrays. The R packages affy and affyPLM from BioConductor [47] were used to establish normalization and NUSE values.

### Normalization of Agilent gene expression data

Level 1 Agilent gene expression data were downloaded from the TCGA for 106 samples for which Infinium 450k DNA methylation data were available. The intensities within array were normalized using Loess normalization, followed by quantile normalization between arrays. Missing values were imputed using nearest neighbor averaging method. In a last step, average intensities were calculated for probes, which are present more than once.

### HOX classification of samples

We used the scaled and centered gene expression data for the 22 and 53 probes measuring levels of HOX-signature genes in the NCH_EORTC and TCGA samples, respectively. These data were used as input for an iterative k-means clustering procedures. Parameters were chosen to search for the most stable cluster consisting of two to eight groups of samples, and 10,000 iterations were performed. The number of groups was selected based on which number of clusters had the maximum Calinksi-Harabasz criterion value [48], thus representing the most stable partitioning of samples into groups/clusters. The mean HOX-signature expression levels were then calculated for the different groups and ‘high’ and ‘low’ classes were assigned based on the observed mean population-wide expression levels (means of the HOX-high/-low sample means).

### Selection of Illumina Infinium 450k probes

Probes measuring DNA methylation of the promoters of 21 HOX signature genes were selected based on their annotated location, resulting in a list of 400 probes. To reduce the dimensionality of the DNA methylation data, principle component analysis was performed and only the 100 probes with the highest cumulative contribution retained. Further details on the procedure are presented in the Extended Experimental Procedures.

### Correlation between gene copy number and expression

Expression data and aCGH profiling were available for 64 GBM samples of the NCH_EORTC cohort. For each of those samples, the median aCGH value and the median gene expression value (after each gene was mean centered and divided by the standard deviation of its expression across those samples) were calculated for each of the 39 autosomal chromosome arms. Pearson correlation coefficients between the median aCGH values and median expression values of all chromosomal arms were calculated per sample as described [17].

### Correlation between DNA methylation and expression

Pearson cross-correlation matrices were computed separately to investigate relationship between the filtered methylation data and HOX expression signature datasets for both NCH_EORTC and TCGA samples. A description of the detailed statistical procedure can be found in the Extended Experimental Procedures.

### Correlation of TCGA GBM Agilent gene and microRNA expression data

The correlation between mean HOX-signature gene expression levels and microRNA expression levels were calculated using Spearman’s rho statistic to estimate rank-based measure of association, for 96 of the 106 TCGA GBM samples. The top 2 percentile positively and negatively correlated microRNAs were selected for further inspection.

### Additive effect of CNA and DNA methylation on mean HOX-signature expression levels

Mean HOX-signature expression level of each sample was calculated from the scaled and centered expression values of each probe (22 for NCH_EORTC, 53 for TCGA). This value was then designated as the response variable of the linear model. The DNA methylation levels of the probe (M-values) were used as the first explanatory variable, and as a second term the CNA levels as determined by circular binary segmentation at cytoband chr7p15.2 were added (log2 fold-change of tumor over diploid reference):

$$ \begin{array}{l} Mean\kern0.5em \left(HOX\kern0.5em  signature\kern0.5em  expression\right)\kern0.5em \sim \kern0.5em DNA\kern0.5em  methylation\kern0.5em \left( selected\kern0.5em 450k\kern0.5em  probe\kern0.5em \right[M\\ {}\kern0.5em \mathit{\hbox{-}}\kern0.5em  value\left]\right)\kern0.5em +\kern0.5em CNA\kern0.5em \left(7p15.2\kern0.5em \left[ logRR\right]\right)\end{array} $$

### Data access

Gene expression profiles, DNA copy number alteration data (array comparative genomic hybridization (aCGH)) and DNA methylation profiles have in part been previously published [7,24,49], and are available in the Gene Expression Omnibus (GEO) database at [50] (accession-number: GSE7696; GSE60507; GSE60274). Due to patient privacy concerns, the RNA-Seq data in the form of raw sequencing data will be made available upon request to the corresponding author, MEH. The molecular profiles of GBM from The Cancer Genome Atlas project (TCGA) were downloaded from [2,51,52].

## Additional files

Additional file 1:
**Figures S1 to** **S15,** **Tables S1 and** **S3 to** **S10 and corresponding figure legends, as well as Extended Experimental Procedures in portable document format.**
Additional file 2: Table S2. Mean correlation with HOX signature expression and *P* values of top 100 selected Infinium 450k probes in NCH_EORTC and TCGA datasets.
