# Supplementary material for: Chromosome 7 gain and DNA hypermethylation at the HOXA10 locus are associated with expression of a stem cell related HOX-signature in glioblastoma
Source: Genome Biol. 2015 Jan 27;16(1):16. doi: 10.1186/s13059-015-0583-7 (PMC4342872; doi:10.1186/s13059-015-0583-7)
Supplement: Additional file 1: — Figures S1 to S15, Tables S1 and S3 to S10 and corresponding figure legends, as well as Extended Experimental Procedures in portable document format. [file 13059_2015_583_MOESM1_ESM.pdf]

Figure S1

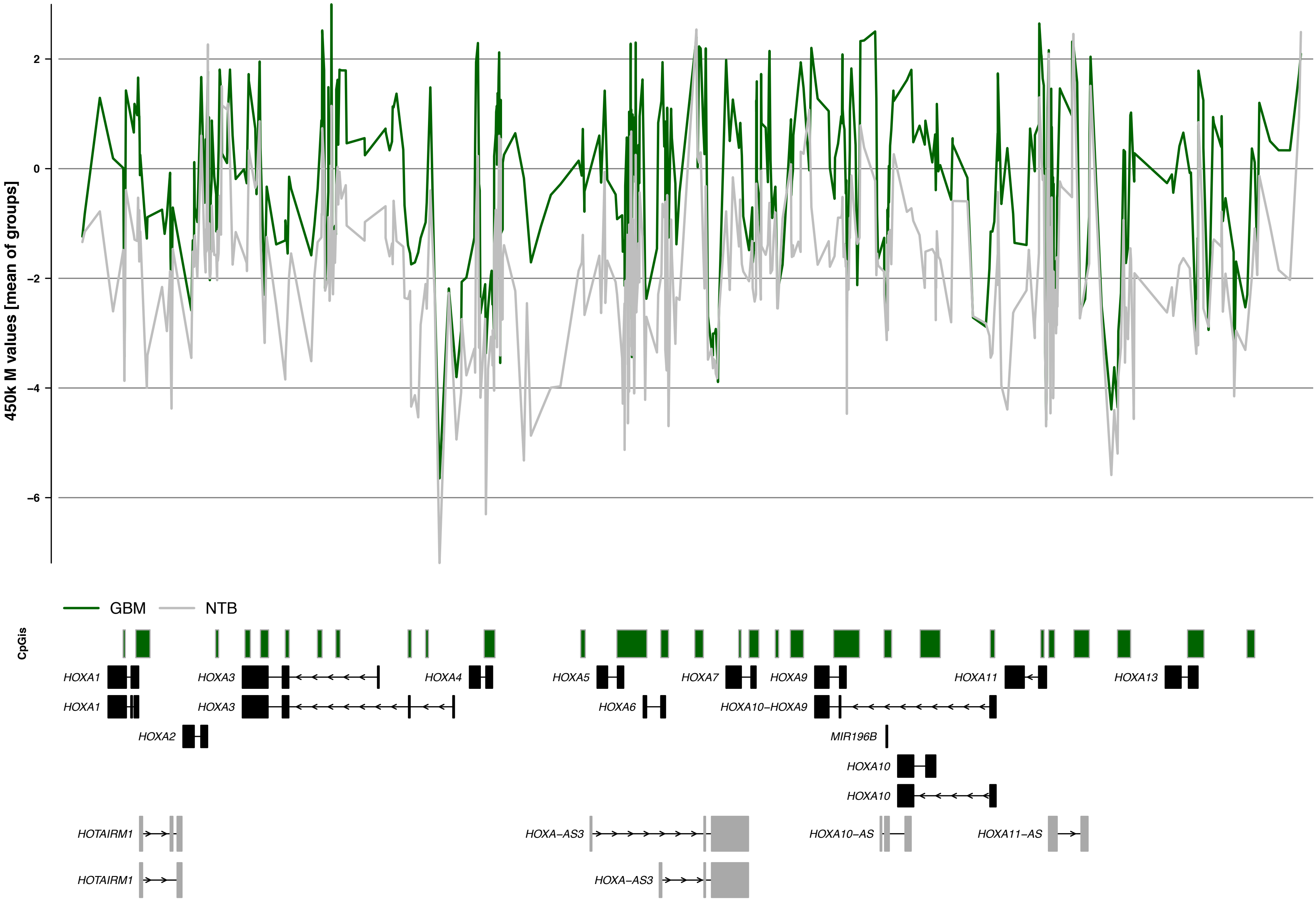

450k M values [mean of groups]
2
0
−2
−4
−6
GBM
NTB
CpGis
HOXA1
HOXA1
HOXA2
HOXA3
HOXA3
HOXA4
HOXA5
HOXA6
HOXA7
HOXA9
HOXA10−HOXA9
MIR196B
HOXA10
HOXA10
HOXA11
HOXA13
HOTAIRM1
HOTAIRM1
HOXA−AS3
HOXA−AS3
HOXA10−AS
HOXA11−AS

Figure S2

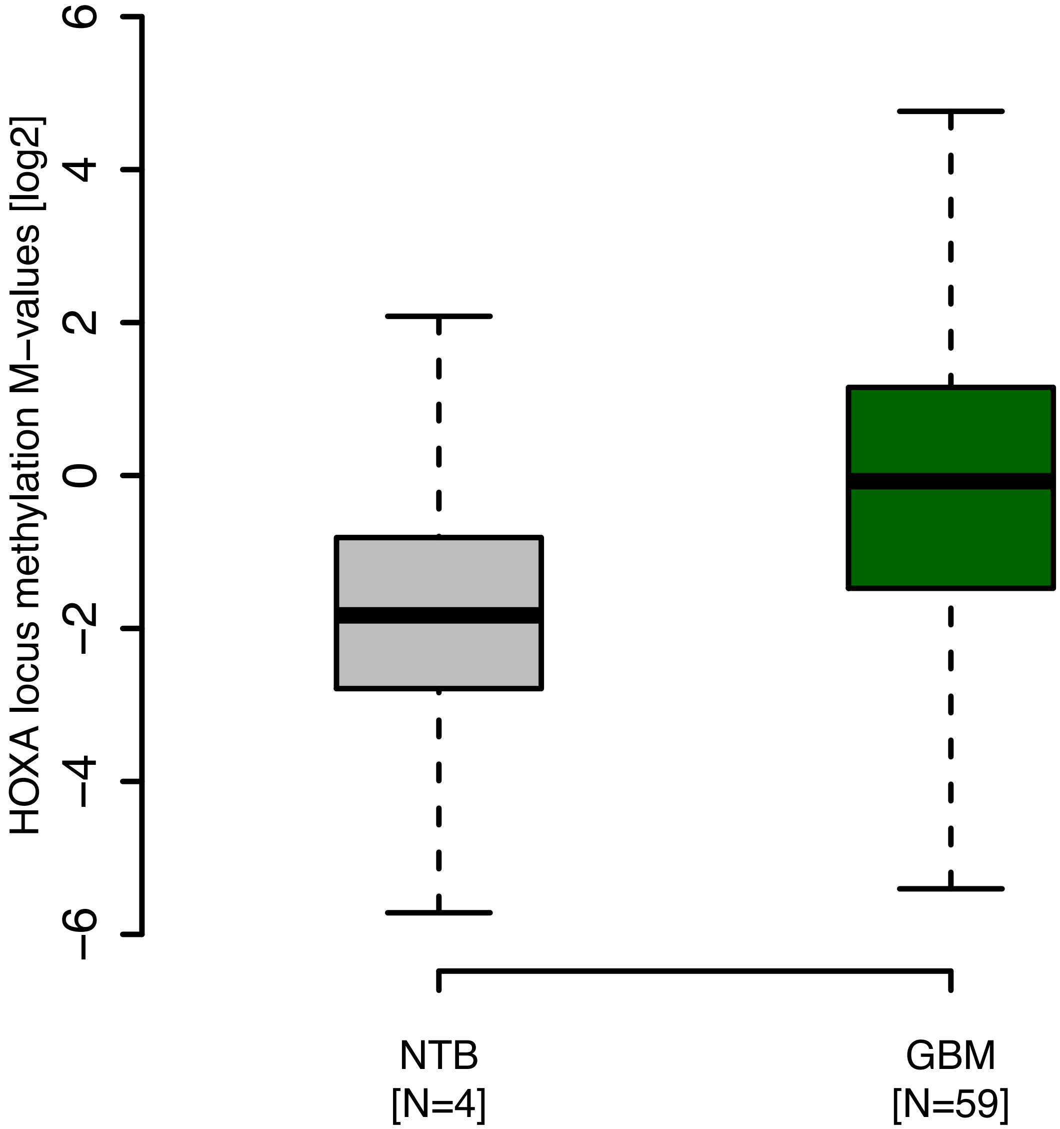

Figure S3

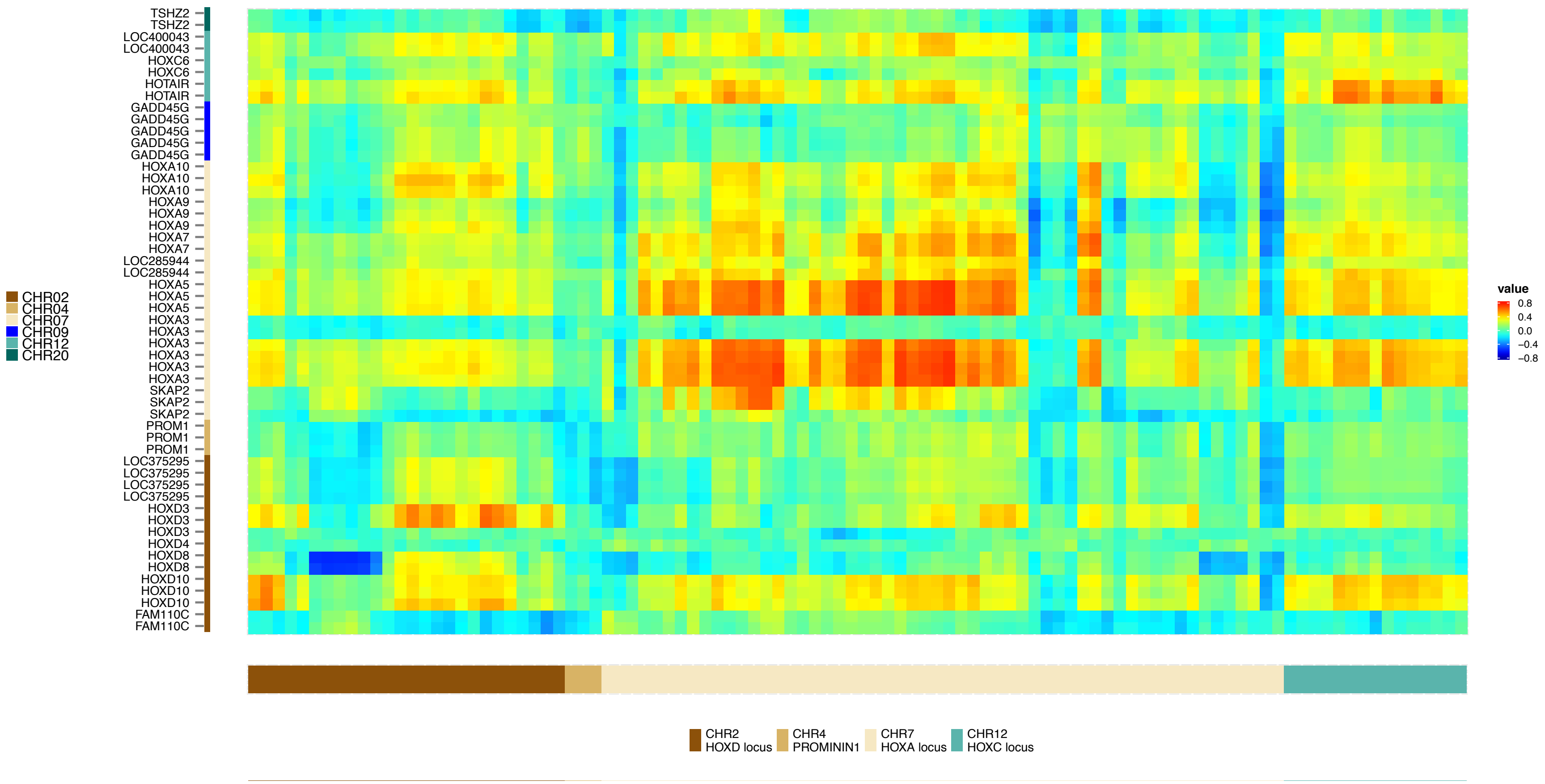
TSHZ2
TSHZ2
LOC400043
LOC400043
HOXC6
HOXC6
HOTAIR
HOTAIR
GADD45G
GADD45G
GADD45G
GADD45G
GADD45G
HOXA10
HOXA10
HOXA10
HOXA9
HOXA9
HOXA9
HOXA7
HOXA7
LOC285944
LOC285944
HOXA5
HOXA5
HOXA5
HOXA3
HOXA3
HOXA3
HOXA3
HOXA3
HOXA3
SKAP2
SKAP2
SKAP2
PROM1
PROM1
PROM1
LOC375295
LOC375295
LOC375295
LOC375295
HOXD3
HOXD3
HOXD3
HOXD4
HOXD8
HOXD8
HOXD10
HOXD10
HOXD10
FAM110C
FAM110C
CHR02
CHR04
CHR07
CHR09
CHR12
CHR20
value
0.8
0.4
0.0
−0.4
−0.8
CHR2 HOXD locus
CHR4 PROMININ1
CHR7 HOXA locus
CHR12 HOXC locus

Figure S4

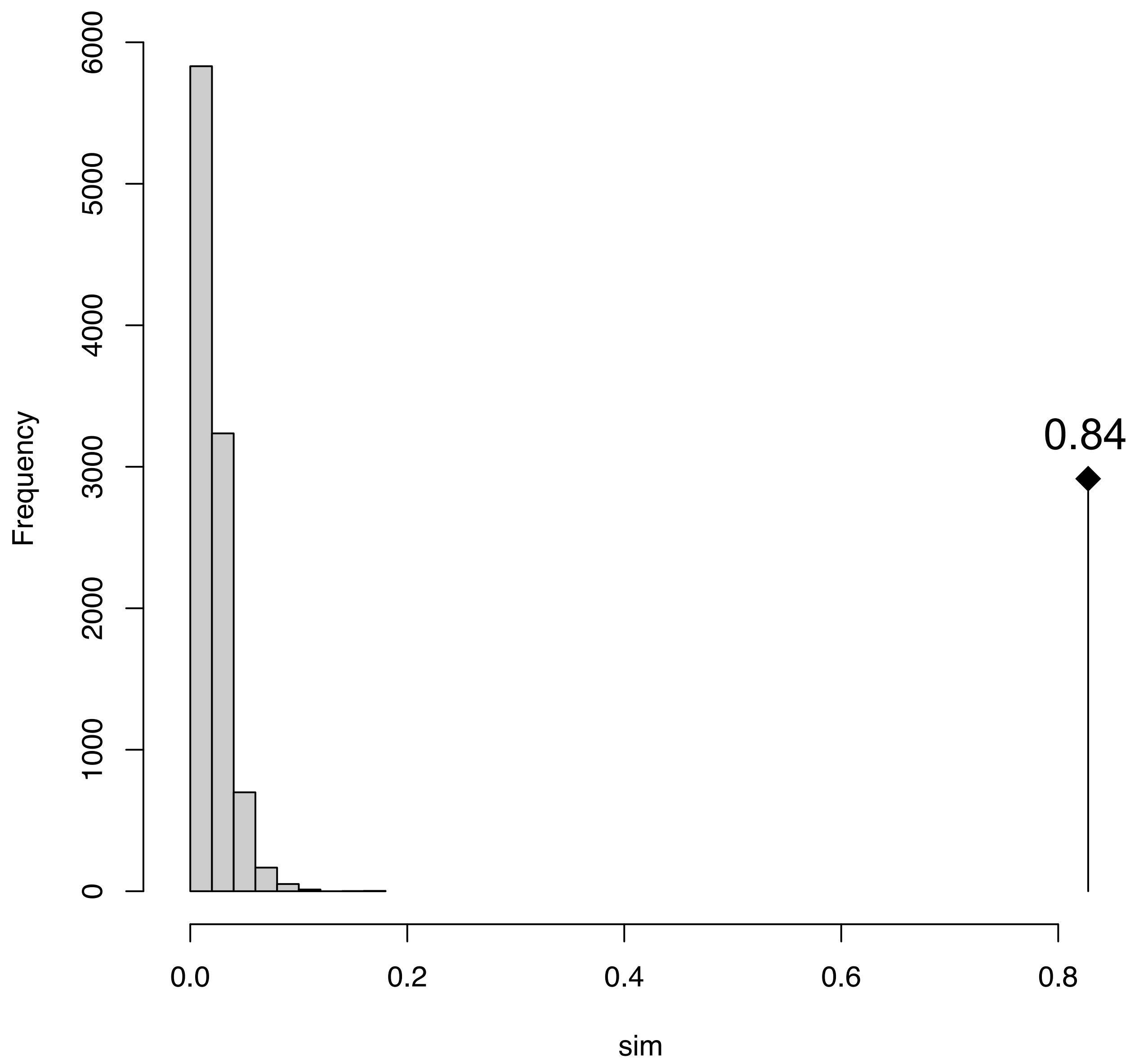

0.84
Frequency
0
1000
2000
3000
4000
5000
6000
0.0
0.2
0.4
0.6
0.8
sim

Figure S5

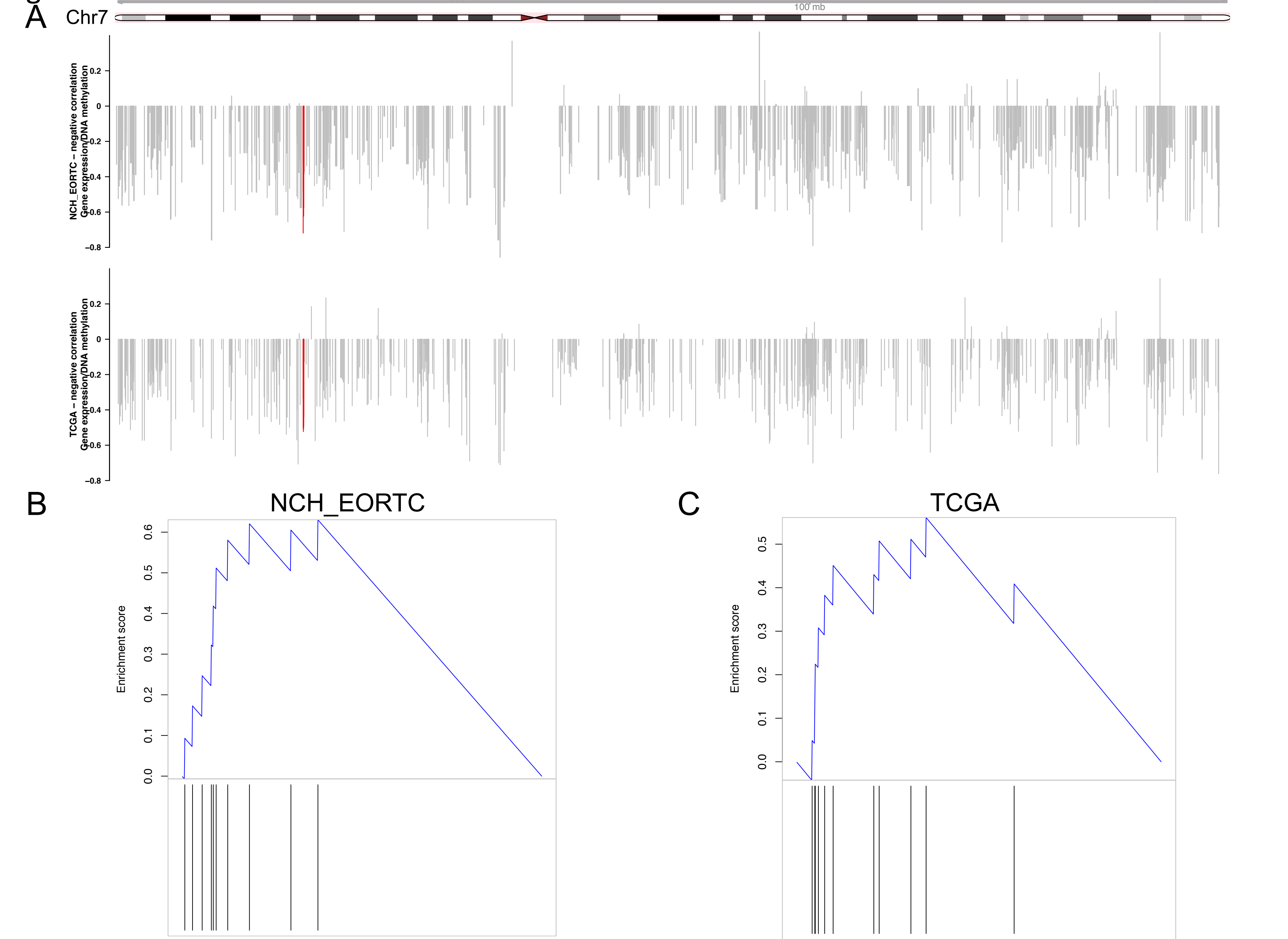
A
Chr7
50 mb
100 mb
150 mb
NCH_EORTC – negative correlation
Gene expression/DNA methylation
TCGA – negative correlation
Gene expression/DNA methylation
0.2
0
-0.2
-0.4
-0.6
-0.8
B
NCH_EORTC
C
TCGA
Enrichment score
0.0
0.1
0.2
0.3
0.4
0.5
0.6

Figure S6

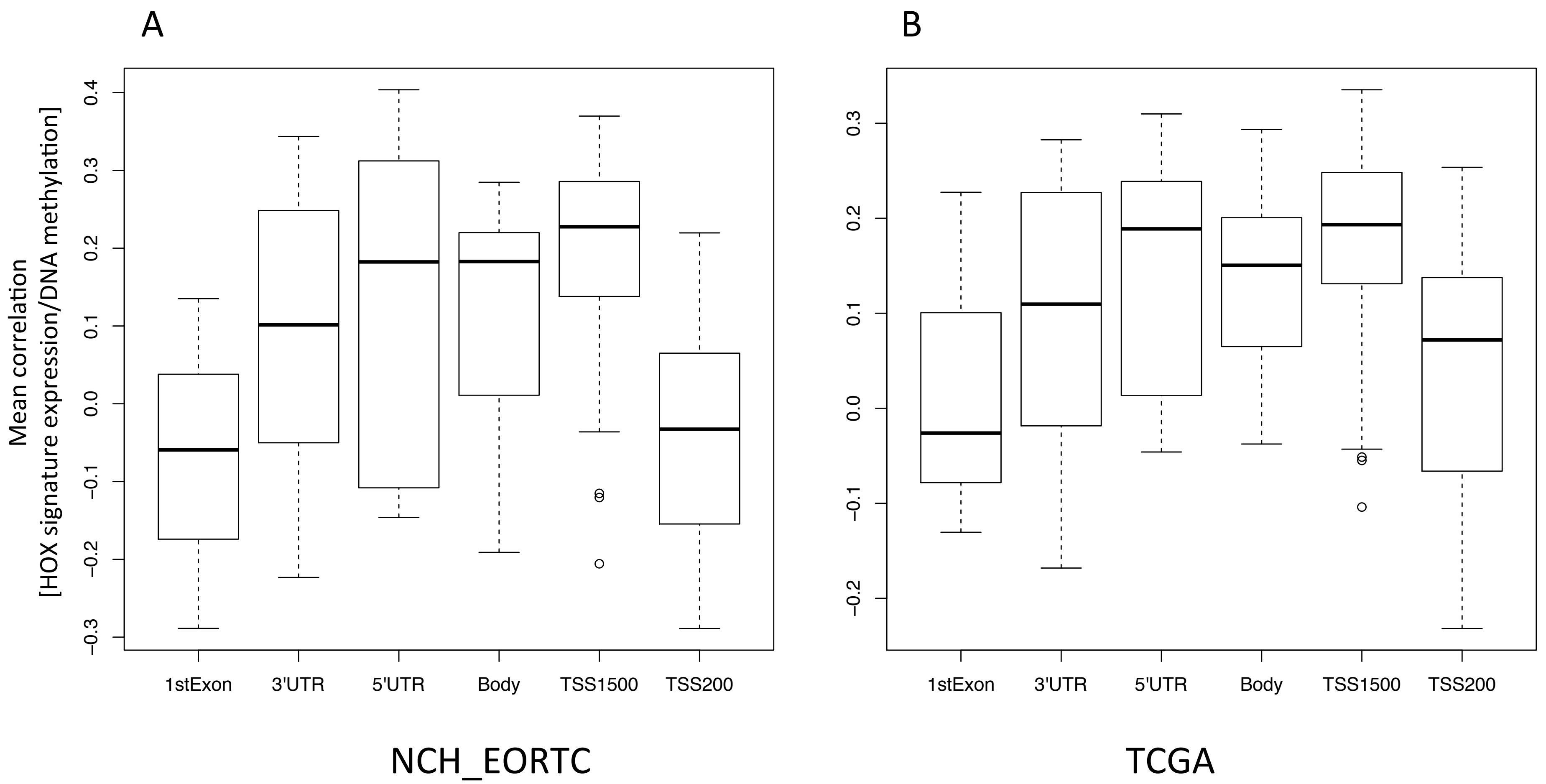
A
B
Mean correlation
[HOX signature expression/DNA methylation]
0.4
0.3
0.2
0.1
0.0
−0.1
−0.2
−0.3
1stExon
3'UTR
5'UTR
Body
TSS1500
TSS200
NCH_EORTC
TCGA

Figure S7

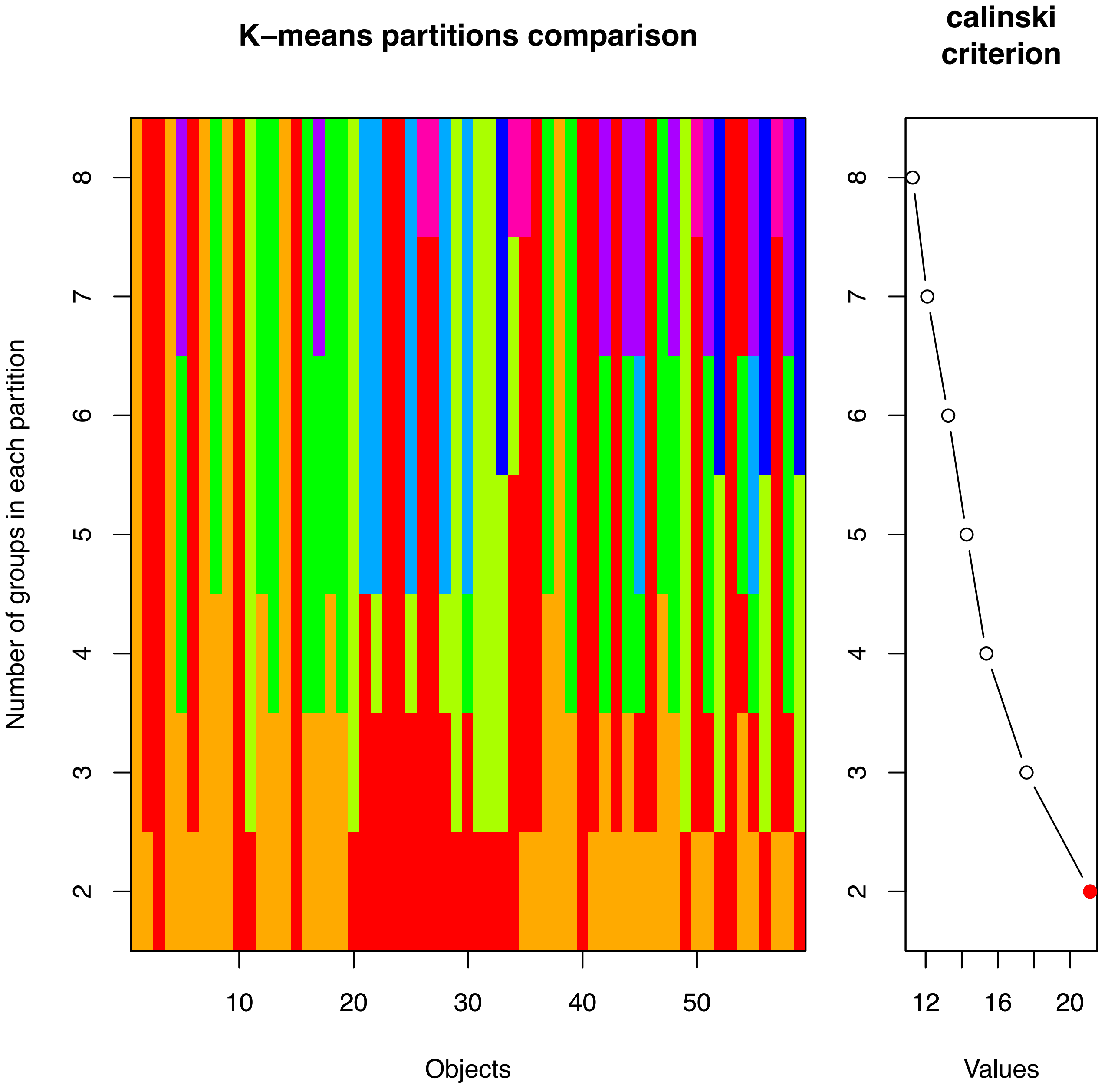

K–means partitions comparison
calinski criterion
Number of groups in each partition
2
3
4
5
6
7
8
10
20
30
40
50
Objects
12
16
20
Values

Figure S8

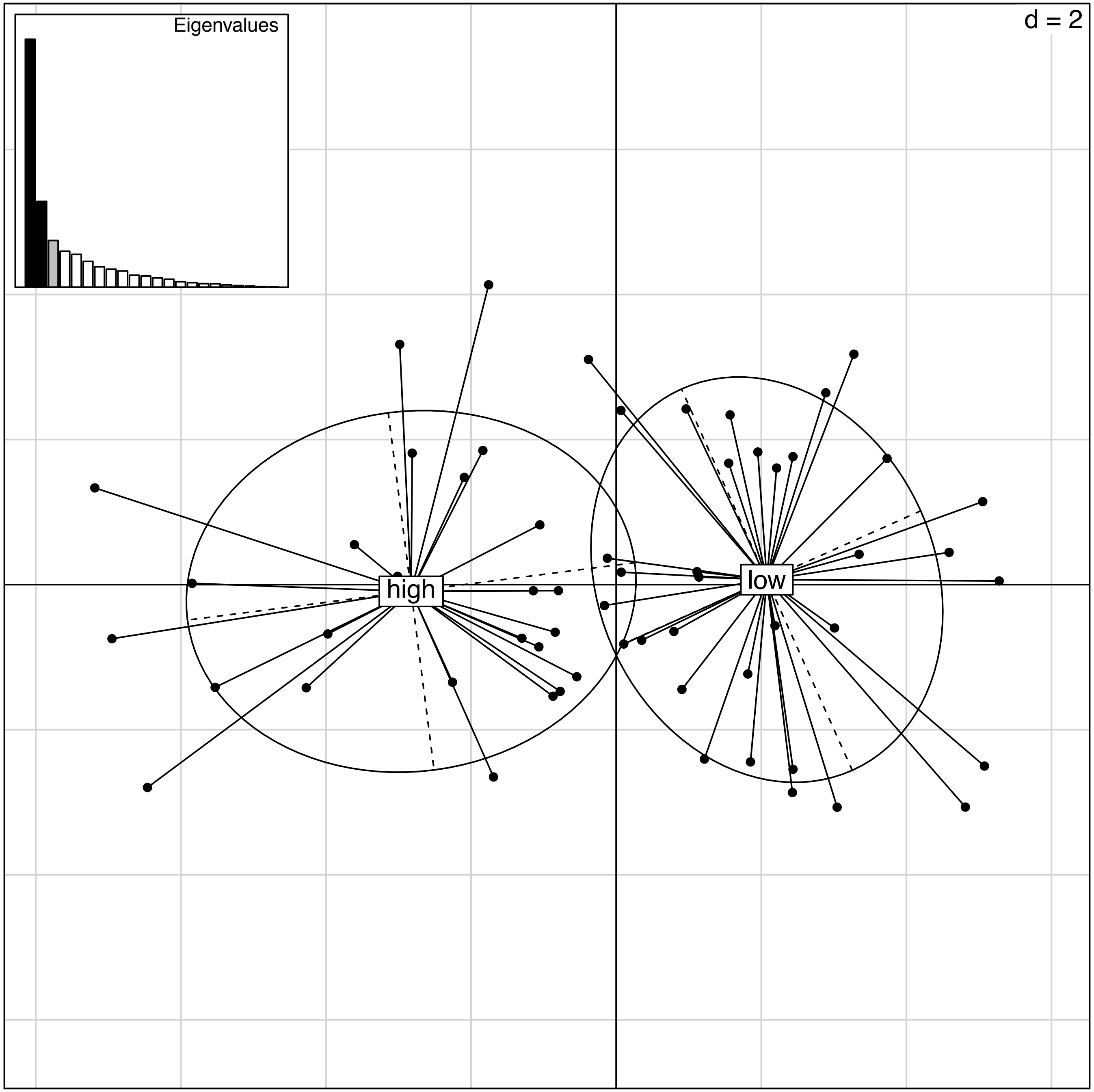

Figure S9

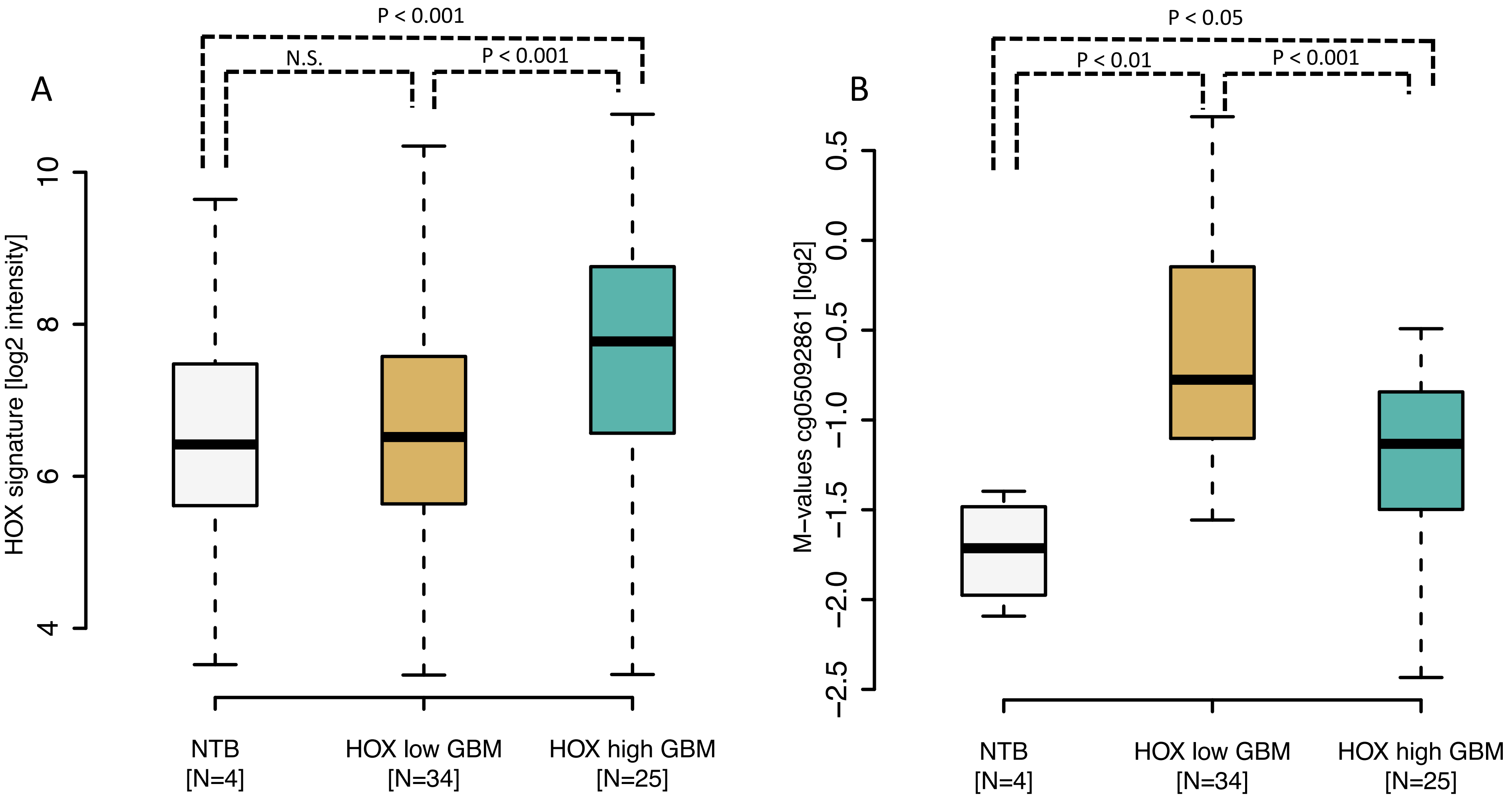
A
P < 0.001
N.S.
P < 0.001
HOX signature [log2 intensity]
4
6
8
10
NTB
[N=4]
HOX low GBM
[N=34]
HOX high GBM
[N=25]
B
P < 0.05
P < 0.01
P < 0.001
M−values cg05092861 [log2]
−2.5
−2.0
−1.5
−1.0
−0.5
0.0
0.5
NTB
[N=4]
HOX low GBM
[N=34]
HOX high GBM
[N=25]

Figure S10

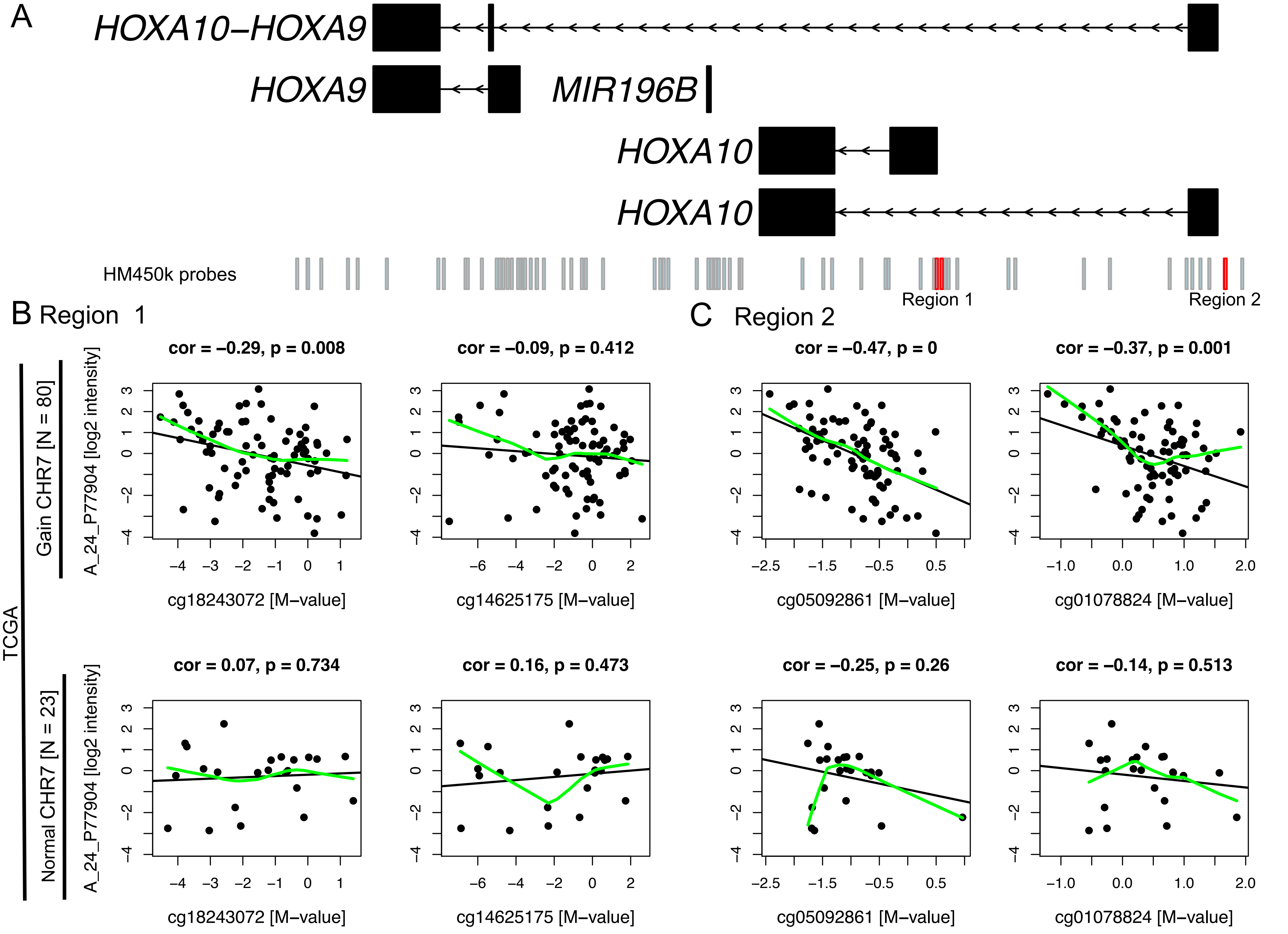
A
HOXA10–HOXA9
HOXA9
MIR196B
HOXA10
HOXA10
HM450k probes
Region 1
Region 2
B Region 1
C Region 2
TCGA
Gain CHR7 [N = 80]
Normal CHR7 [N = 23]
A_24_P77904 [log2 intensity]
cor = –0.29, p = 0.008
cor = –0.09, p = 0.412
cor = –0.47, p = 0
cor = –0.37, p = 0.001
cor = 0.07, p = 0.734
cor = 0.16, p = 0.473
cor = –0.25, p = 0.26
cor = –0.14, p = 0.513
cg18243072 [M–value]
cg14625175 [M–value]
cg05092861 [M–value]
cg01078824 [M–value]

Figure S11

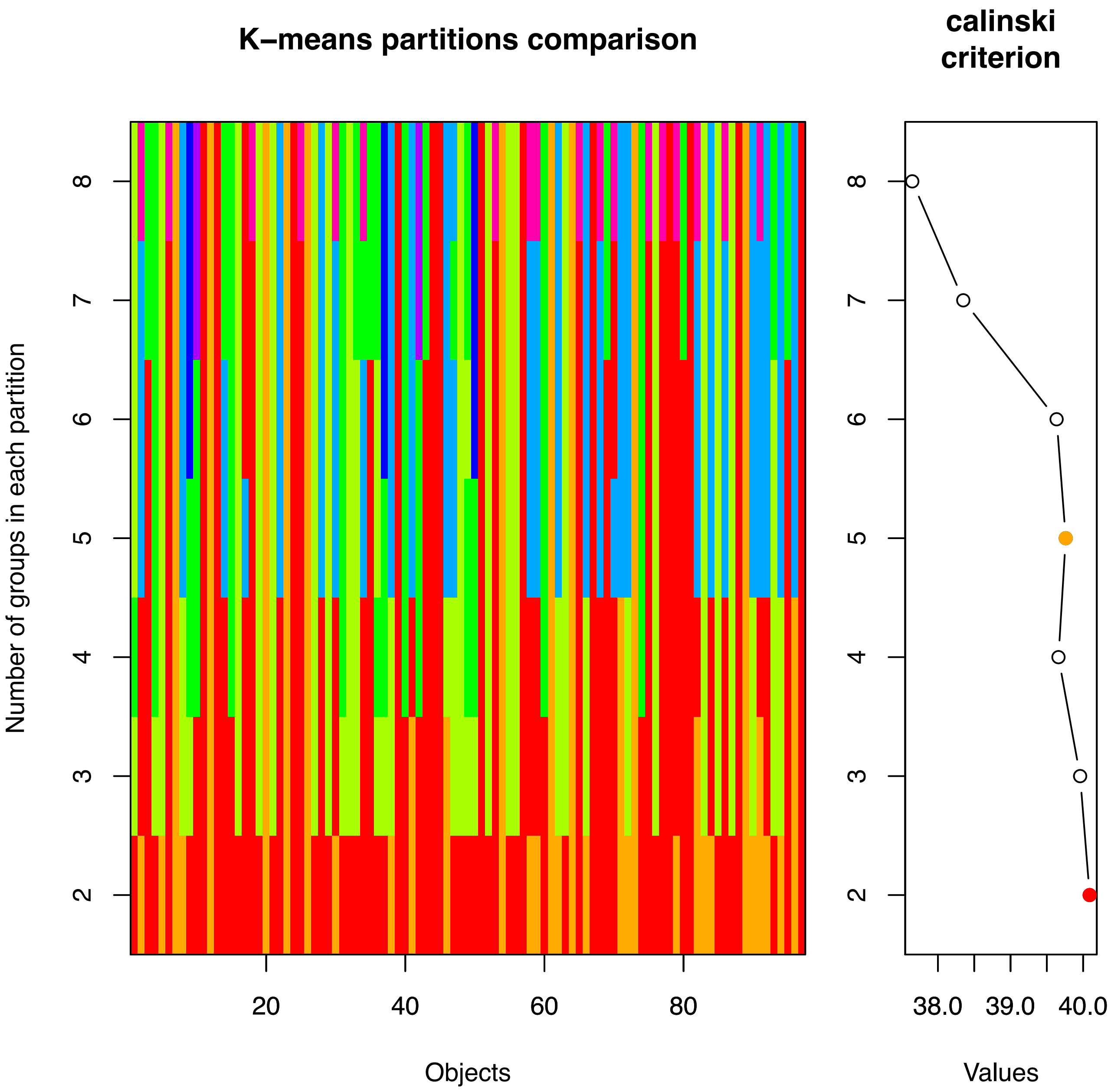

K–means partitions comparison
calinski criterion
Number of groups in each partition
8
7
6
5
4
3
2
20
40
60
80
Objects
38.0
39.0
40.0
Values

Figure S12

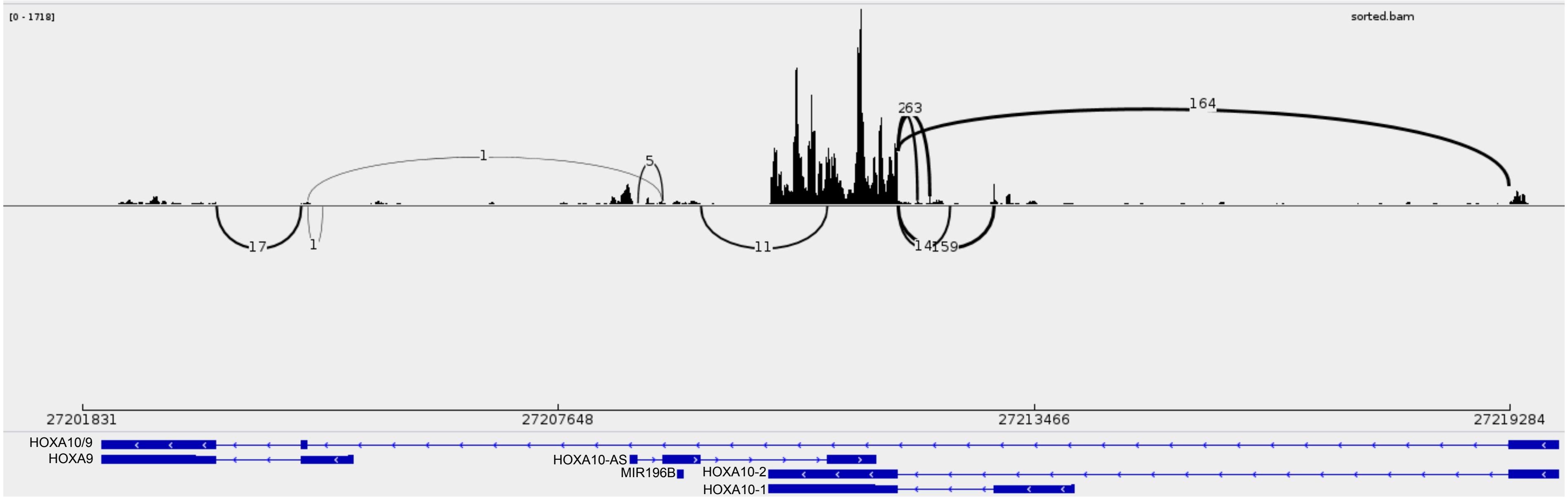

[0 - 1718]
sorted.bam
1
5
263
164
17
1
11
14
159
27201831
27207648
27213466
27219284
HOXA10/9
HOXA9
HOXA10-AS
MIR196B
HOXA10-2
HOXA10-1

Figure S13

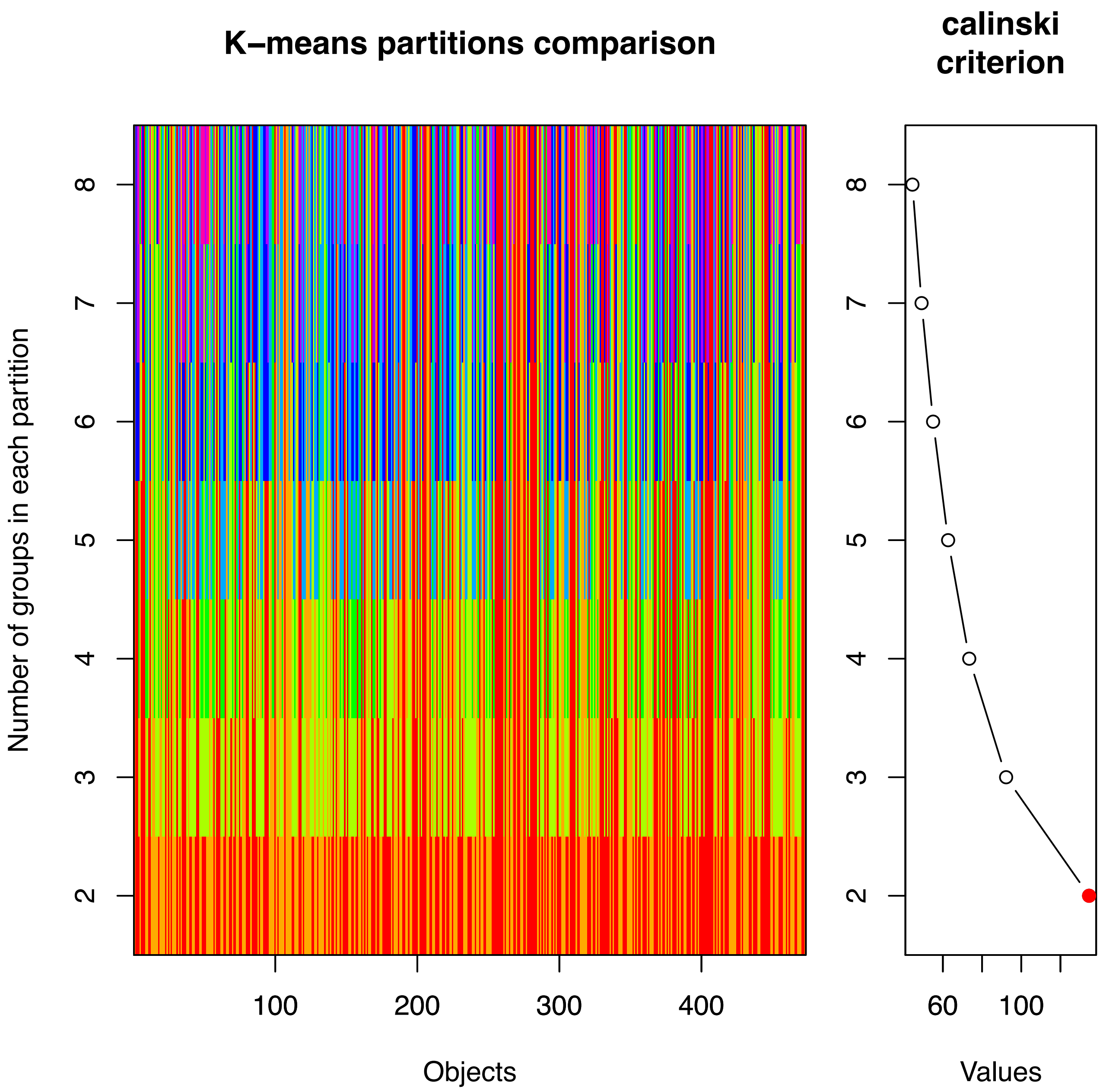
K–means partitions comparison
calinski
criterion
Number of groups in each partition
8
7
6
5
4
3
2
100
200
300
400
Objects
60
100
Values

Figure S14

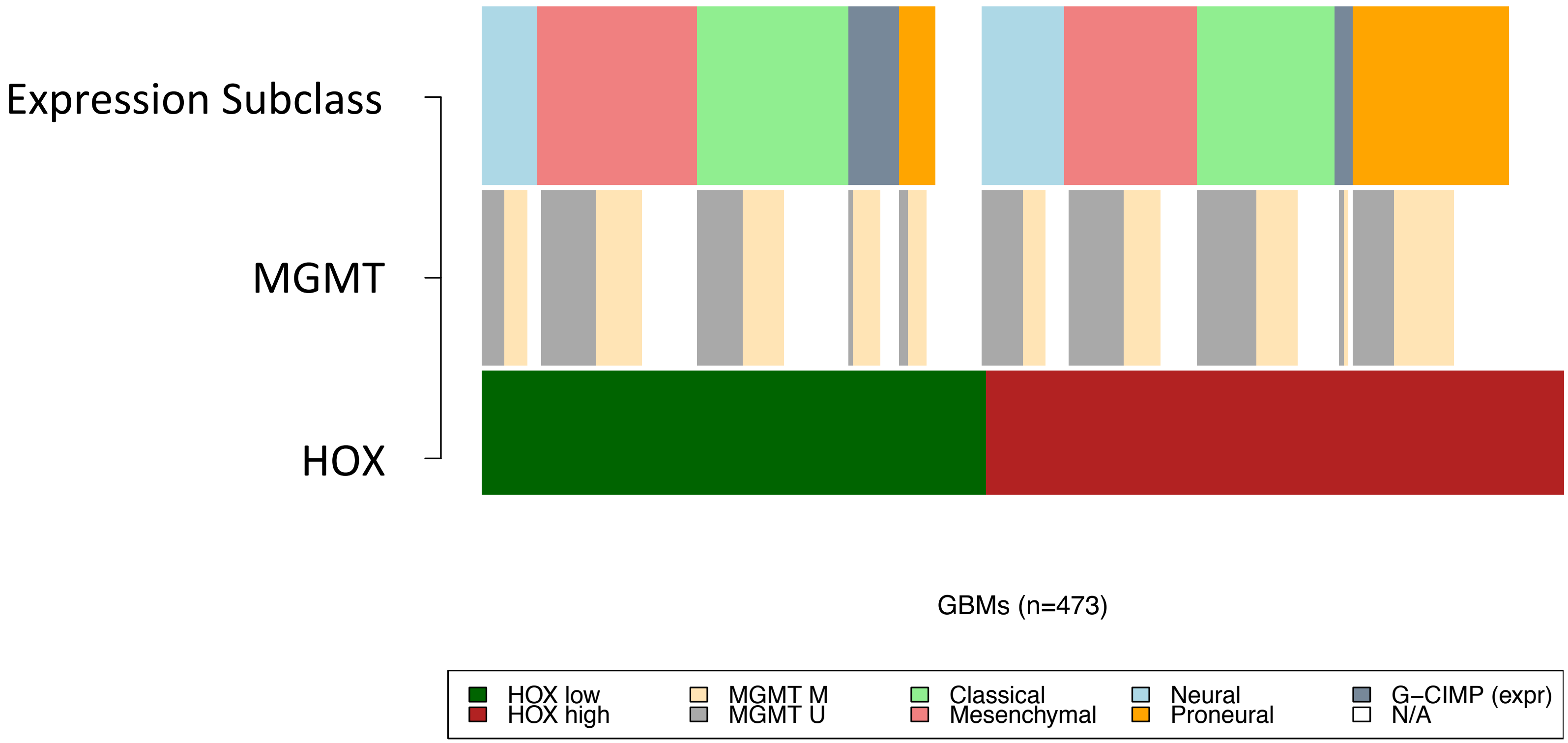
Expression Subclass
MGMT
HOX
GBMs (n=473)
HOX low
HOX high
MGMT M
MGMT U
Classical
Mesenchymal
Neural
Proneural
G–CIMP (expr)
N/A

Figure S15

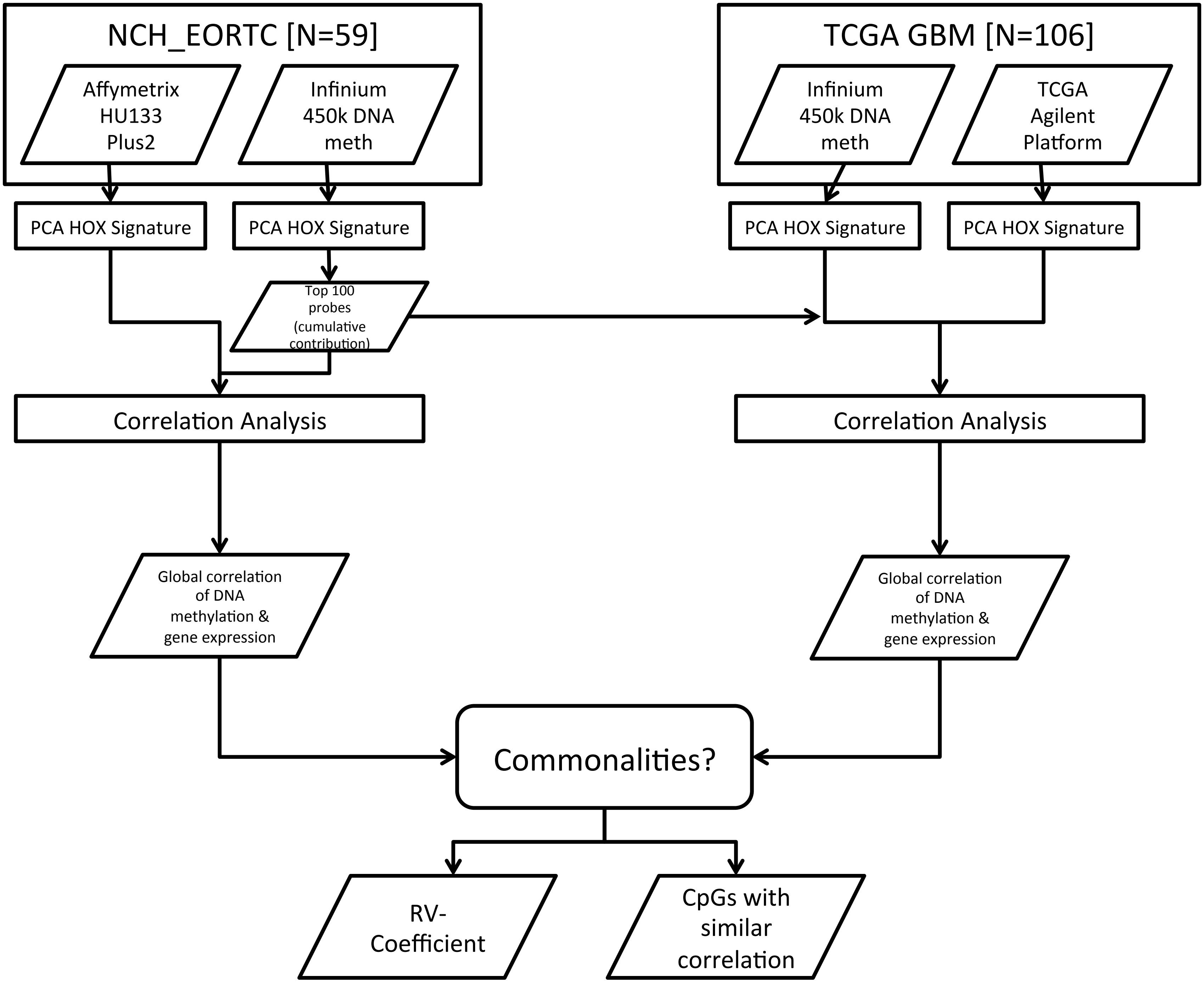
NCH_EORTC [N=59]
Affymetrix HU133 Plus2
Infinium 450k DNA meth
PCA HOX Signature
PCA HOX Signature
Top 100 probes (cumulative contribution)
Correlation Analysis
Global correlation of DNA methylation & gene expression
TCGA GBM [N=106]
Infinium 450k DNA meth
TCGA Agilent Platform
PCA HOX Signature
PCA HOX Signature
Correlation Analysis
Global correlation of DNA methylation & gene expression
Commonalities?
RV-Coefficient
CpGs with similar correlation

SUPPLEMENTARY FIGURES LEGENDS

Figure S1. Pattern of DNA methylation across the *HOXA* locus in brain tissue and GBM.

Mean M-values, for 4 non-tumoral brain (NTB) samples (grey) and 59 GBM (dark green, NCH_EORTC cohort) are plotted for the 504 CpGs located in the *HOXA* locus on chromosome 7 (27,130,000 to 27,250,000; hg19 UCSC) available on the Illumina Infinium 450k DNA methylation BeadChip. Corresponding RefSeq annotation and UCSC CpG islands of the *HOXA* locus are plotted at the bottom.

Figure S2. Comparison of mean DNA methylation of the *HOXA* locus in brain and GBM. Related to Figure S1.

Boxplot of M-values for 4 non-tumoral brain (NTB) samples and 59 GBM (NCH_EORTC cohort) measured at 504 CpGs in the *HOXA* locus on CHR7. GBM are hypermethylated as compared to brain (p-value < 0.001, two-sided t-test, outliers have been omitted for clarity).

Figure S3. Correlation between expression of HOX-signature and DNA methylation. Related to Figure 1.

The heatmap visualizes the correlation between selected top 100 450k probes measuring DNA methylation of CpGs (x-axis) and the expression of 19 HOX-signature genes measured by 53 Agilent probes (y-axis) in 106 GBMs from TCGA.

Figure S4. Similarity between correlation matrix of HOX-signature gene expression / DNA methylation in test-and validation–set. Related to Figure 1A and S3.

The RV-coefficient was determined by bootstrapping for the correlation matrices of the HOX-signature gene expression / DNA methylation data-sets of the NCH_EORTC and TCGA cohorts. The RV-coefficient, which measures the relative similarity of the two matrices was statistically significant (RV= 0.84, simulated p-value < 0.001 [9999 permutations]) and confirms the high similarity between the observed correlation patterns.

Figure S5. Negative correlation between gene expression and DNA methylation for NCH_EORTC and TCGA GBM samples plotted across chromosome 7 and accompanying gene set enrichment analysis. Related to Figure 1.

The strongest negative correlations between DNA methylation probes and CHR7 genes were plotted according to genomic locations of the respective genes. NCH_EORTC and TCGA data are shown separately in panel **A**. Highlighted in red are the correlation coefficients of 10 and 11 *HOXA* genes, respectively. Panel **B** illustrates the positions of the 10 *HOXA* genes against the CHR7-wide background of 711 genes ranked by correlation coefficient. The enrichment of minimum correlation coefficients for the *HOXA* genes is statistically significant (GSEA, p-value < 0.001). Panel **C** shows the results of GSEA for TCGA data. *HOXA* genes are also significantly enriched against the CHR7 background of 791 genes (p-value < 0.001).

Figure S6. Location specific correlation patterns between HOX-signature expression and DNA methylation. Related to Figures 1A and S3.

Mean correlation of the top 100 450k probes in function of their annotated probe locations for NCH_EORTC (**A**) and TCGA (**B**) data. Overall, probes with a more negative mean correlation - indicative of canonical effects of DNA methylation on gene expression - are observed for probes annotated to be located in the 1st exon and within 200bp of the transcription start site (TSS).

Figure S7. Clustering of NCH_EORTC GBM using expression of HOX-signature genes results in two stable groups. Related to Figure 3A.

Diagnostic plots of k-means clustering of 59 NCH_EORTC samples using HOX-signature genes. Colored bars in the rows represent the cluster-membership of each of the 59 samples. The line plot illustrates the Calinski-criterion values for each configuration of clusters. The red dot represents the configuration with the highest Calinski-criterion and thus the most stable organization of samples into different clusters. In the HOX-signature based clustering of NCH_EORTC samples, two clusters represent the most stable organization.

Figure S8. Principle component analysis of HOX-signature expression levels confirms that observed variability in expression distinguishes two groups. Related to Figures 3A and S6.

Plot of first two principle components (PC1 on x-axis, PC2 on y-axis) of 59 NCH_EORTC GBM based on HOX-signature gene expression. GBM classified by k-means clustering into HOX-high and HOX-low are effectively separated by the first two principle components. Eigenvalues of the PCA are shown in the inserted bar plot.

Figure S9. HOX-signature expression levels and DNA methylation of Infinium 450k probe cg05092861 compared between NTB and HOX high and low GBMs. Related to Figures 3A, S9 and S10.

A Box-plot of mean expression of levels of HOX-signature genes in 4 NTB samples and 59 GBMs classified as HOX-low (34) and HOX-high (25). Mean expression levels in HOX-low samples are not different to expression levels NTB (p-value > 0.94), while expression in HOX-high samples is significantly different to both HOX-low (p-value < 0.001) and NTB (p-value < 0.001, all pairwise comparisons using Welch Two Sample t-test). **B** M-values of the top 450k probe negatively correlated with HOX-signature expression levels measured in NTB, HOX-low and HOX-high GBM. The difference in DNA methylation between the three groups was assessed using pairwise tests (Welch Two Sample t-test) and showed that there is a small but statistically significant difference between NTB and HOX-high GBM- (p-value < 0.05). Differences in DNA methylation between HOX-low GBM and NTB (p-value < 0.01) and HOX-high GBM (p-value < 0.001) are more pronounced and also statistically significant.

Figure S10. Correlation of HOXA10 expression and DNA methylation at the canonical and alternative promoter in 103 TCGA samples, stratified by CHR7 status. Related to Figures 3 & 4.

To illustrate the relationship between gene expression and DNA methylation, X-Y plots of M-values and log2 intensities of *HOXA10* associated probes are shown. For visual orientation, an overview of the *HOXA10* genomic region is shown in panel **A** (based on RefSeq annotation, retrieved from Ensembl using reference genome hg19/GRCh37). Additionally, the location of Illumina Infinium 450k probes have been plotted; highlighted in red are the four probes which have been used to generate the X-Y plots in panels **B** & **C**. The top row of panels **B** and **C** shows the expression and DNA methylation of samples with a trisomy of

CHR7 (N = 80). Bottom row of panels **B** and **C** show the corresponding data from samples with normal CHR7 status (N = 23). Pearson's product moment correlation coefficient *cor* and p-values are shown above each plot. Black lines in the plots show the fit of linear regression, in green local regression using lowess smoothing is shown.

Figure S11. Clustering of GBM using expression of HOX-signature genes results in two stable groups in TCGA GBM samples with matched Agilent expression and Illumina Infinium 450k data. Related to Figure 3B.

Diagnostic plots of k-means clustering of 106 TCGA samples using HOX-signature genes. The line plot illustrates the Calinski-criterion values for each configuration of clusters. The red dot represents the configuration with the highest Calinski-criterion and thus the most stable organization of samples into two different clusters.

Figure S12. Visualization of mapped RNA-Seq reads to a region on CHR7 covering *HOXA10* and *HOXA9* genes. Related to Figure 5.

Sashimi plot of paired-end RNA-Seq reads from four glioma sphere lines aligned to the human *HOXA10-9* region on chromosome 7 (bp 27200752-27220726, hg19). The connecting arcs between the different clusters of reads illustrate the presence of cDNA fragments which captures splice junctions, and thus support the finding of a potential presence of a read-through transcript which includes exons of *HOXA9*, the ncRNA RP1170O19.20, *miR-196b*, and *HOXA10*.

Figure S13. Clustering of 473 TCGA GBM samples using expression levels of HOX-signature genes into two stable groups,

Diagnostic plots of k-means clustering of 473 TCGA samples using level 2 data (probe-level normalized) of 19 HOX-signature genes. The line plot illustrates the Calinski-criterion values for each configuration of clusters. The red dot represents the configuration with the highest Calinski-criterion and thus the most stable organization of samples is into two different clusters.

Figure S14. Association of HOX-high/low with molecular GBM subclassifications of TCGA samples. Related to Figure S13 and Tables S7 and S8.

The 473 GBM samples from TCGA were classified into HOX-high (n = 259) and HOX-low (n = 214) as described in methods. Annotations for expression subtype (Neural, Proneural, Mesenchymal, Classical), G-CIMP, and *MGMT* promoter methylation status were taken from Brennan et al (Brennan et al. 2013). The color code is explained in the Figure. The HOX-high GBM are enriched in proneural subtype, while they are underrepresented in the proneural G-CIMP-positive subtype (p-value <0.001, Pearson's Chi-squared test, see Table S6).

Figure S15. Illustration of the data flow used in the presented analysis.

Flowchart of the coinertia analysis used to identify CpGs potentially involved in regulation of *HOX* signature gene expression.

Table S1**:** List of 22 Affymetrix HU133Plus2 probes measuring expression levels of HOX signature transcripts of 21 genes

Probe Set ID	Gene Symbol	Gene Title	Alignments
226863_at	FAM110C	family with sequence similarity 110, member C	CHR2:38813-42283 (-)
238847_at	HOXD10	HOXD10	CHR2:176975838-176976565 (+)
229400_at	HOXD10	homeobox D10	CHR2:176983967-176984669 (+)
231906_at	HOXD8	homeobox D8	CHR2:176994429-176997422 (+)
205522_at	HOXD3 /// HOXD4 /// MIR10B	homeobox D3 /// homeobox D4 /// microRNA 10b	CHR2:177015117-177020798 (+)
228564_at	LOC375295	hypothetical LOC375295	CHR2:177494317-177502302 (-)
204304_s_at	PROM1	prominin 1	CHR4:15969856-16077566 (-)
225639_at	SKAP2	src kinase associated phosphoprotein 2	CHR7:26706686-26708299 (-)
204362_at	SKAP2	src kinase associated phosphoprotein 2	CHR7:26708300-26904211 (-)
228642_at	HOTAIRM1	HOTAIRM1	CHR7:27135851-27140019 (+)
1557051_s_at	HOTAIRM1	HOTAIRM1	CHR7:27136047-27139585 (+)
235521_at	HOXA3	homeobox A3	CHR7:27146626-27147537 (-)
213844_at	HOXA5	homeobox A5	CHR7:27180687-27183283 (-)
235753_at	HOXA7	homeobox A7	CHR7:27193339-27194232 (-)
209905_at	HOXA10 /// HOXA9	homeobox A10 /// homeobox A9	CHR7:27202056-27205149 (-)
214651_s_at	HOXA10 /// HOXA9	homeobox A10 /// homeobox A9	CHR7:27202219-27209269 (-)
213150_at	HOXA10	homeobox A10	CHR7:27210223-27214191 (-)
204121_at	GADD45G	growth arrest and DNA-damage-inducible, gamma	CHR9:92219927-92221467 (+)
239153_at	HOTAIR	hox transcript antisense RNA (non-protein coding)	CHR12:54356091-54356560 (-)
206858_s_at	HOXC6	homeobox C6	CHR12:54422193-54424520 (+)
226582_at	LOC400043	hypothetical LOC400043	CHR12:54519845-54526624 (+)
244521_at	TSHZ2	teashirt zinc finger homeobox 2	CHR20:52108144-52108623 (+)

Table S3: Gene-Set Enrichment Analysis of negative correlations between gene expression and DNA methylation across chromosome 7

Dataset	Minimum negative correlations [counts]		GSEA statistics			
	Chr7-wide	HOXA genes	+ (enriched)	p-value	- (depleted)	p-value
NCH_EORTC	746	10	0.6304	<0.0001	-0.0068	0.9919
TCGA	816	11	0.5613	0.0004	-0.0422	0.9397

Table S4: List of TCGA GBM samples with Agilent gene expression, Illumina Infinium 450k DNA methylation and Agilent SNP6 CNV data used in the analysis

TCGA-19-0957	TCGA-74-6584	TCGA-06-5411
TCGA-19-1389	TCGA-06-6694	TCGA-76-6193
TCGA-06-0152	TCGA-26-6173	TCGA-06-6390
TCGA-14-1402	TCGA-14-1395	TCGA-76-6282
TCGA-76-4928	TCGA-32-1980	TCGA-19-5952
TCGA-06-5417	TCGA-76-6286	TCGA-06-6701
TCGA-28-5215	TCGA-26-1442	TCGA-19-5955
TCGA-76-4925	TCGA-74-6578	TCGA-06-6388
TCGA-76-4932	TCGA-76-6285	TCGA-14-1043
TCGA-76-4929	TCGA-19-5958	TCGA-28-2501
TCGA-06-5416	TCGA-76-6283	TCGA-74-6575
TCGA-28-5209	TCGA-06-5413	TCGA-76-6660
TCGA-12-5299	TCGA-06-6695	TCGA-15-1444
TCGA-28-5219	TCGA-76-6657	TCGA-74-6581
TCGA-32-5222	TCGA-19-5956	TCGA-41-6646
TCGA-76-4927	TCGA-06-6693	TCGA-76-6191
TCGA-28-5220	TCGA-06-0650	TCGA-41-5651
TCGA-28-5213	TCGA-28-2510	TCGA-76-6656
TCGA-26-5139	TCGA-26-6174	TCGA-06-5856
TCGA-76-4935	TCGA-76-6662	TCGA-19-5947
TCGA-26-5133	TCGA-06-5408	
TCGA-76-4931	TCGA-06-6698	
TCGA-26-5135	TCGA-06-1804	
TCGA-28-5214	TCGA-28-6450	
TCGA-12-5295	TCGA-76-6280	
TCGA-28-5208	TCGA-76-6664	
TCGA-28-5218	TCGA-14-0862	
TCGA-28-5216	TCGA-81-5911	
TCGA-06-5415	TCGA-81-5910	
TCGA-26-5136	TCGA-06-6389	
TCGA-28-5204	TCGA-14-0781	
TCGA-76-4926	TCGA-87-5896	
TCGA-12-5301	TCGA-76-6661	
TCGA-26-5132	TCGA-06-5410	
TCGA-06-5418	TCGA-19-5959	
TCGA-76-4934	TCGA-74-6577	
TCGA-26-5134	TCGA-19-5954	
TCGA-06-5414	TCGA-28-5211	
TCGA-28-5207	TCGA-06-6699	
TCGA-06-6391	TCGA-19-5953	
TCGA-14-0740	TCGA-06-6700	
TCGA-06-5859	TCGA-06-6697	
TCGA-14-1450	TCGA-06-5412	

Table S5: TCGA Agilent 244k probe IDs of HOX signature genes

	Reporter ID	Composite Gene SymbolEntrez Gene ID	Composite Coordinates
1	A_24_P50248	FAM110C|642273	[36.3:2:31608-36385:-]
2	A_24_P50250	FAM110C|642273	[36.3:2:31608-36385:-]
3	A_23_P131640	HOXD10|3236	[36.3:2:176689738-176692916:+]
4	A_23_P381368	HOXD10|3236	[36.3:2:176689738-176692916:+]
5	A_23_P381366	HOXD10|3236	[36.3:2:176689738-176692916:+]
6	A_23_P210164	HOXD8|3234	[36.3:2:176702723-176704974:+]
7	A_24_P188651	HOXD8|3234	[36.3:2:176702723-176704974:+]
8	A_23_P345725	HOXD4|3233	[36.3:2:176724359-176726197:+]
9	A_23_P79652	HOXD3|3232	[36.3:2:176737051-176746072:+]
10	A_23_P323181	HOXD3|3232	[36.3:2:176737051-176746072:+]
11	A_23_P323180	HOXD3|3232	[36.3:2:176737051-176746072:+]
12	A_32_P16196	LOC375295|375295	[36.3:2:177202555-177263956:-]
13	A_23_P302781	LOC375295|375295	[36.3:2:177202555-177263956:-]
14	A_23_P302787	LOC375295|375295	[36.3:2:177202555-177263956:-]
15	A_32_P16204	LOC375295|375295	[36.3:2:177202555-177263956:-]
16	A_24_P234658	PROM1|8842	[36.3:4:15578955-15686664:-]
17	A_23_P258462	PROM1|8842	[36.3:4:15578955-15686664:-]
18	A_23_P258463	PROM1|8842	[36.3:4:15578955-15686664:-]
19	A_24_P920333	SKAP2|8935	[36.3:7:26673212-26870866:-]
20	A_23_P157127	SKAP2|8935	[36.3:7:26673212-26870866:-]
21	A_23_P157128	SKAP2|8935	[36.3:7:26673212-26870866:-]
22	A_23_P501536	HOXA3|3200	[36.3:7:27112334-27133164:-]
23	A_23_P501538	HOXA3|3200	[36.3:7:27112334-27133164:-]
24	A_32_P198180	HOXA3|3200	[36.3:7:27112334-27133164:-]
25	A_32_P198179	HOXA3|3200	[36.3:7:27112334-27133164:-]
26	A_23_P111571	HOXA3|3200	[36.3:7:27112334-27133164:-]
27	A_23_P111572	HOXA3|3200	[36.3:7:27112334-27133164:-]
28	A_23_P93769	HOXA5|3202	[36.3:7:27147521-27149812:-]
29	A_23_P93772	HOXA5|3202	[36.3:7:27147521-27149812:-]
30	A_23_P93773	HOXA5|3202	[36.3:7:27147521-27149812:-]
31	A_24_P829209	LOC285944|285944,HOXA7|3204	[36.3:7:27159860-27162821:-]
32	A_24_P829201	LOC285944|285944,HOXA7|3204	[36.3:7:27159860-27162821:-]
33	A_23_P70968	HOXA7|3204,LOC285944|285944	[36.3:7:27159860-27162821:-]
34	A_23_P70972	HOXA7|3204,LOC285944|285944	[36.3:7:27159860-27162821:-]
35	A_23_P500998	HOXA9|3205	[36.3:7:27168582-27171674:-]
36	A_23_P334930	HOXA9|3205	[36.3:7:27168582-27171674:-]
37	A_23_P215384	HOXA9|3205	[36.3:7:27168582-27171674:-]
38	A_24_P77904	HOXA10|3206	[36.3:7:27176735-27186368:-]
39	A_23_P253368	HOXA10|3206	[36.3:7:27176735-27186368:-]
40	A_23_P253364	HOXA10|3206	[36.3:7:27176735-27186368:-]
41	A_23_P157950	GADD45G|10912	[36.3:9:91409748-91411290:+]

Table S6: Correlation between gene expression and DNA methylation of HOXA10 and Infinium 450k probes located in HOXA10 CGI for NCH_EORTC and TCGA samples, stratified by CHR7 status.

		NCH_EORTC				TCGA					
		Gain CHR7 [N = 38]		Normal CHR7 [N = 21]		Gain CHR7 [N = 80]		Normal CHR7 [N = 23]			
UCSC CpGi	HM450k probe ID	cor	p-value	cor	p-value	cor	p-value	cor	p-value	Relation to CpGi	Comment
chr7:27212416-27214396 (Region 1 in Figures 4 & S8)	cg07483304	-0.38	0.02	0.08	0.726	-0.24	0.033	0.25	0.244	N_Shore	
	cg05490659	-0.5	0.001	-0.02	0.945	-0.32	0.004	0.04	0.839	N_Shore	
	cg03987115	-0.36	0.028	-0.24	0.302	-0.33	0.003	-0.13	0.541	Island	
	cg19351701	-0.33	0.041	-0.19	0.401	-0.2	0.08	-0.1	0.665	Island	
	**cg13703049**	**-0.5**	**0.001**	**-0.11**	**0.645**	**-0.26**	**0.019**	**0**	**0.986**	**Island**	**Part of 100 selected HM450k probes**
	**cg21172377**	**-0.46**	**0.003**	**0.08**	**0.729**	**-0.27**	**0.016**	**-0.03**	**0.897**	**Island**	**Part of 100 selected HM450k probes**
	**cg18243072**	**-0.4**	**0.014**	**-0.19**	**0.42**	**-0.29**	**0.008**	**0.07**	**0.734**	**Island**	**Part of 100 selected HM450k probes**
	cg01397139	-0.22	0.188	-0.15	0.51	-0.04	0.719	0.14	0.522	Island	
	cg16967880	-0.28	0.084	-0.22	0.328	-0.07	0.527	0.27	0.213	Island	
	cg16857858	-0.27	0.097	0.12	0.603	0	0.98	0.07	0.764	Island	
	cg18416576	-0.32	0.051	0.2	0.376	-0.08	0.492	0.13	0.541	Island	
	**cg14625175**	**-0.41**	**0.01**	**0.16**	**0.495**	**-0.09**	**0.412**	**0.16**	**0.473**	**Island**	**Part of 100 selected HM450k probes**
	cg01215762	-0.35	0.029	0.22	0.347	-0.01	0.935	0.13	0.548	Island	
	cg14649140	-0.2	0.237	0.16	0.48	0.03	0.793	0.14	0.535	Island	
	**cg09411999**	**-0.07**	**0.695**	**0.01**	**0.968**	**0.17**	**0.141**	**0.26**	**0.23**	**Island**	**Part of 100 selected HM450k probes**
	cg14935646	0.06	0.72	0.21	0.371	0.47	0	0.36	0.094	S_Shore	
	cg27157482	0.05	0.745	0.11	0.637	0.37	0.001	0.5	0.016	S_Shore	
	cg02483701	0.2	0.232	0.29	0.204	0.45	0	0.5	0.016	S_Shore	
chr7:27219309-27219750 (Region 2 in Figures 4 & S8)	cg09636715	-0.6	0	-0.2	0.382	-0.37	0.001	-0.18	0.399	N_Shelf	
	cg15864691	-0.56	0	-0.09	0.706	-0.57	0	-0.28	0.201	N_Shore	
	cg10724867	-0.42	0.009	0.16	0.5	-0.18	0.115	0.06	0.774	N_Shore	
	cg00870179	-0.27	0.106	-0.23	0.315	-0.27	0.015	-0.27	0.219	Island	
	cg17047659	-0.45	0.004	-0.11	0.628	-0.39	0	-0.11	0.629	Island	
	**cg05092861**	**-0.52**	**0.001**	**-0.01**	**0.974**	**-0.47**	**0**	**-0.25**	**0.26**	**S_Shore**	**Part of 100 selected HM450k probes**
	**cg01078824**	**-0.49**	**0.002**	**-0.12**	**0.603**	**-0.37**	**0.001**	**-0.14**	**0.513**	**S_Shore**	**Part of 100 selected HM450k probes**
	cg05517976	-0.44	0.006	-0.02	0.929	-0.47	0	0.02	0.941	S_Shore	
	cg14188840	-0.13	0.439	0	0.986	0.31	0.006	0.47	0.025	S_Shore	
	cg08575233	-0.33	0.041	-0.05	0.836	-0.25	0.026	0.33	0.127	S_Shore	

Table S7: Correlation between HOX signature mean expression and DNA methylation of selected 100 Illumina Infnium 450k probes, stratified by CHR7 status.

		NCH_EORTC				TCGA					
		Trisomy CHR7 [N = 38]		Normal CHR7 [N = 21]		Trisomy CHR7 [N = 80]		Normal CHR7 [N = 23]			
UCSC CpGi	HM450k probe	cor	p-value	cor	p-value	cor	p-value	cor	p-value	Relation to CpGi	Comment
chr2:176980765-176981423	cg20649017	0.21	0.214	0.57	0.007	0.35	0.002	0.55	0.007	Island	
	cg10364040	0.13	0.429	0.65	0.001	0.42	0	0.67	0	Island	
chr2:176982107-176982402	cg25953239	0.27	0.1	0.58	0.006	0.45	0	0.46	0.025	Island	
chr2:176992950-176993186	cg00035316	-0.28	0.088	0.36	0.114	-0.12	0.272	0.35	0.099	Island	
	cg18448949	-0.23	0.171	0.65	0.001	0.17	0.122	0.48	0.022	Island	
chr2:176993479-176995557	cg03858756	-0.48	0.003	0.28	0.222	-0.08	0.485	-0.01	0.952	Island	
	cg15808943	-0.46	0.004	0.48	0.029	-0.05	0.658	-0.03	0.91	Island	
	cg24416513	-0.39	0.016	0.44	0.045	0.05	0.682	-0.09	0.689	Island	
	cg15520279	-0.4	0.013	0.37	0.095	-0.01	0.918	-0.05	0.819	Island	
	cg10239098	-0.32	0.054	0.43	0.054	-0.12	0.294	0.03	0.895	Island	
	cg01293179	-0.16	0.349	0.35	0.125	-0.05	0.66	0.28	0.195	S_Shore	
chr2:177014948-177015214	cg24000528	0.12	0.472	0.01	0.972	0.17	0.133	0.52	0.011	N_Shore	
	cg23460578	0.31	0.057	0.08	0.744	0.43	0	0.53	0.01	N_Shore	
	cg22352800	0.26	0.119	0.19	0.415	0.53	0	0.58	0.004	N_Shore	
	cg12127282	0.33	0.045	0.14	0.55	0.45	0	0.62	0.002	N_Shore	
	cg08717880	0.21	0.196	0.15	0.523	0.46	0	0.6	0.003	N_Shore	
	cg01152019	0.27	0.097	0.11	0.646	0.43	0	0.57	0.004	Island	
chr2:177017266-177017489	cg10803577	0.12	0.476	0.27	0.245	0.37	0.001	0.44	0.036	N_Shore	
chr2:177016416-177016632	cg09701582	0.16	0.328	0.5	0.02	0.45	0	0.49	0.018	S_Shore	
chr2:177029413-177029941	cg01128482	0.27	0.095	0.27	0.242	0.53	0	0.63	0.001	N_Shore	
	cg10304824	0.2	0.228	0.07	0.776	0.47	0	0.62	0.002	N_Shore	
	cg24541426	0.16	0.342	0.32	0.154	0.4	0	0.53	0.01	Island	
	cg05864326	-0.33	0.041	-0.04	0.858	0.04	0.712	0.46	0.026	S_Shore	
chr2:177036254-177037213	cg01171212	0.17	0.301	0.31	0.176	0.27	0.017	0.4	0.057	Island	
	cg04316624	0.25	0.138	0.12	0.591	0.31	0.005	0.52	0.012	Island	
chr2:177039551-177039951	cg13053653	0.02	0.892	0.35	0.12	-0.06	0.605	0.28	0.199	N_Shore	
chr4:16084195-16085735	cg07656173	-0.05	0.755	-0.44	0.044	-0.09	0.447	-0.14	0.524	S_Shore	
	cg13117948	0.05	0.764	-0.53	0.013	-0.01	0.963	0.08	0.727	S_Shore	
	cg08855742	-0.25	0.135	-0.35	0.12	-0.11	0.31	-0.13	0.554	S_Shore	
chr7:27146069-27146600	cg12538674	-0.24	0.142	0.63	0.002	-0.01	0.933	0.34	0.109	N_Shore	
chr7:27147589-27148389	cg27539480	-0.63	0	0.37	0.098	-0.41	0	0.07	0.762	N_Shore	
	cg01175550	-0.36	0.026	0.52	0.015	-0.21	0.061	0.49	0.018	N_Shore	
	cg13240116	-0.16	0.329	0.72	0	0.29	0.01	0.7	0	N_Shore	
	cg07153966	-0.23	0.165	0.71	0	0.17	0.121	0.63	0.001	Island	

		NCH_EORTC				TCGA					
		Trisomy CHR7 [N = 38]		Normal CHR7 [N = 21]		Trisomy CHR7 [N = 80]		Normal CHR7 [N = 23]			
UCSC CpGi	HM450k probe	cor	p-value	cor	p-value	cor	p-value	cor	p-value	Relation to CpGi	Comment
chr7:27150030-27150418	cg02693607	-0.24	0.144	0.57	0.007	0.34	0.002	0.63	0.001	S_Shore	
chr7:27153187-27153647	cg21134232	0.07	0.655	0.67	0.001	0.4	0	0.66	0.001	S_Shore	
	cg01027532	0.04	0.829	0.7	0	0.29	0.01	0.6	0.003	S_Shore	
	cg02000808	0.01	0.933	0.56	0.009	0.13	0.256	0.41	0.054	S_Shore	
chr7:27154999-27155426	cg12305431	0.34	0.037	0.59	0.005	0.58	0	0.55	0.007	S_Shelf	
	cg26297005	0.6	0	0.67	0.001	0.54	0	0.68	0	Island	
	cg18430152	0.52	0.001	0.5	0.022	0.52	0	0.57	0.004	Island	
	cg19240213	0.67	0	0.6	0.004	0.63	0	0.53	0.01	N_Shore	
chr7:27163819-27164098	cg14573448	0.48	0.002	0.59	0.005	0.39	0	0.43	0.042	S_Shore	
	cg00524179	0.27	0.105	0.69	0.001	0.49	0	0.58	0.004	N_Shelf	
	cg14974749	0.18	0.292	0.5	0.02	0.15	0.196	0.42	0.048	N_Shore	
	cg20974609	0.15	0.354	0.31	0.165	0.24	0.034	0.58	0.003	N_Shore	
	cg19196335	0.18	0.276	0.44	0.048	0.32	0.003	0.56	0.006	Island	
	cg16997642	0.03	0.878	0.37	0.101	0.11	0.326	0.54	0.008	Island	
	cg20817131	0.26	0.109	0.45	0.04	0.16	0.149	0.54	0.008	Island	
	cg14013695	0.32	0.049	0.5	0.021	0.46	0	0.64	0.001	Island	
chr7:27182613-27185562	cg01323381	0.5	0.001	0.4	0.076	0.56	0	0.53	0.009	Island	
	cg05774699	0.45	0.005	0.54	0.012	0.53	0	0.71	0	Island	
	cg26023912	0.29	0.072	0.3	0.186	0.26	0.021	0.61	0.002	Island	
	cg00969405	0.37	0.022	0.49	0.024	0.53	0	0.59	0.003	Island	
	cg03368099	0.5	0.001	0.46	0.037	0.51	0	0.76	0	Island	
	cg01748892	0.55	0	0.53	0.014	0.56	0	0.79	0	Island	
	cg13694927	0.47	0.003	0.54	0.011	0.66	0	0.75	0	Island	
	cg03744763	0.47	0.003	0.21	0.35	0.63	0	0.74	0	Island	
	cg08396193	0.32	0.05	0.58	0.006	0.42	0	0.57	0.005	N_Shore	
	cg24389054	0.47	0.003	0.56	0.008	0.56	0	0.59	0.003	N_Shore	
chr7:27194583-27194827	cg21778348	0.21	0.2	0.34	0.127	0.58	0	0.74	0	Island	
	cg16764637	0.21	0.212	0.44	0.048	0.62	0	0.62	0.002	Island	
	cg08248516	0.32	0.048	0.2	0.389	0.62	0	0.62	0.002	Island	
chr7:27195601-27196567	cg11910375	0.25	0.129	0.34	0.135	0.36	0.001	0.65	0.001	N_Shore	
	cg27508551	-0.53	0.001	-0.31	0.172	-0.29	0.008	-0.09	0.679	Island	
	cg08934785	-0.27	0.095	-0.15	0.514	-0.17	0.136	0.13	0.569	N_Shore	
	cg26511321	-0.18	0.276	0.01	0.954	-0.09	0.415	0.21	0.331	N_Shore	
chr7:27198182-27198514	cg00599770	-0.45	0.004	-0.16	0.49	-0.25	0.025	-0.07	0.744	N_Shore	
	cg15372603	0.43	0.008	0.42	0.061	0.52	0	0.64	0.001	N_Shore	
	cg02000318	0.54	0	0.32	0.159	0.6	0	0.64	0.001	N_Shore	

UCSC CpGi	HM450k probe	NCH_EORTC Trisomy CHR7 [N = 38] cor	NCH_EORTC Trisomy CHR7 [N = 38] p-value	NCH_EORTC Normal CHR7 [N = 21] cor	NCH_EORTC Normal CHR7 [N = 21] p-value	TCGA Trisomy CHR7 [N = 80] cor	TCGA Trisomy CHR7 [N = 80] p-value	TCGA Normal CHR7 [N = 23] cor	TCGA Normal CHR7 [N = 23] p-value	Relation to CpGi	Comment
	cg03217995	-0.37	0.021	-0.38	0.092	-0.05	0.667	0.36	0.09	N_Shore	
	cg21007852	-0.31	0.06	-0.22	0.333	-0.15	0.193	0.26	0.225	N_Shore	
	cg20741169	0.01	0.974	0.42	0.055	0.28	0.01	0.37	0.086	Island	
chr7:27203915-27206462	cg18447772	-0.15	0.378	0.09	0.695	0.24	0.034	0.36	0.089	Island	
	cg12600174	-0.21	0.198	0.02	0.938	0.12	0.298	0.3	0.159	Island	
	cg22055728	-0.15	0.366	0.06	0.789	0.18	0.112	0.4	0.057	Island	
	cg21942490	0.05	0.773	0.18	0.443	0.33	0.003	0.65	0.001	Island	
	cg02643054	0.1	0.547	0.58	0.006	0.36	0.001	0.64	0.001	S_Shore	
	cg13703049	-0.51	0.001	0	0.992	-0.14	0.221	0.06	0.776	Island	
	cg21172377	-0.46	0.004	0.12	0.595	-0.15	0.173	0.2	0.357	Island	
chr7:27212416-27214396	cg18243072	-0.32	0.047	-0.15	0.517	-0.19	0.091	0.15	0.503	Island	Region 1 in Figures 4A & S8A
	cg14625175	-0.37	0.023	0.2	0.393	0.02	0.834	0.24	0.264	Island	Region 1 in Figures 4A & S8A
	cg09411999	0.05	0.75	0.25	0.284	0.34	0.002	0.43	0.04	Island	
chr7:27219309-27219750	cg05092861	-0.64	0	-0.08	0.718	-0.53	0	-0.2	0.369	S_Shore	Region 2 in Figures 4A & S8A
	cg01078824	-0.52	0.001	-0.06	0.784	-0.43	0	-0.2	0.358	S_Shore	Region 2 in Figures 4A & S8A
	cg15731655	0.22	0.175	0.55	0.009	0.35	0.001	0.55	0.006	N_Shelf	
chr12:54359658-54359906	cg06622953	0.2	0.239	0.64	0.002	0.25	0.026	0.58	0.004	N_Shore	
	cg14691529	0.21	0.213	0.25	0.267	0.19	0.095	0.51	0.013	N_Shore	
chr12:54366815-54369103	cg02371798	0.27	0.107	0.71	0	0.25	0.023	0.43	0.04	N_Shelf	
	cg04105511	0.35	0.032	0.62	0.003	0.46	0	0.51	0.013	S_Shore	
	cg26019295	0.42	0.008	0.44	0.045	0.54	0	0.52	0.011	S_Shore	
	cg18040878	0.38	0.02	0.43	0.05	0.51	0	0.44	0.034	S_Shore	
	cg15660418	0.13	0.435	0.33	0.144	0.37	0.001	0.49	0.019	S_Shore	
	cg01473837	0.42	0.009	0.62	0.003	0.45	0	0.54	0.007	S_Shore	
chr12:54408426-54408713	cg22621272	0.31	0.061	0.52	0.015	0.42	0	0.49	0.017	S_Shore	
	cg16937769	0.32	0.051	0.62	0.003	0.35	0.002	0.52	0.011	S_Shore	
	cg04704531	0.19	0.251	0.62	0.003	0.36	0.001	0.47	0.025	S_Shore	
	cg06714180	0.25	0.133	0.61	0.003	0.36	0.001	0.4	0.055	S_Shore	
	cg15817960	0.23	0.165	0.47	0.032	0.27	0.017	0.45	0.032	S_Shore	
	cg13726459	0.09	0.598	0.56	0.009	0.21	0.062	0.41	0.05	S_Shore	

Table S8: Organization of expression subtypes in TCGA GBM HOX-high/low samples

	Classical	G-CIMP	Mesenchymal	Neural	Proneural	N/A
HOX-low [n=214]	66	23	69	25	16	7
HOX-high [n=259]	61	8	57	37	68	6

Table S9: MGMT promoter methylation status in TCGA HOX-high/low samples

	Methylated	Unmethylated	N/A
HOX-low [n=219]	66	62	91
HOX-high [n=254]	73	87	94

Table S10: Primers used for methylation-specific clone sequencing and ChIP-qPCR of *HOXA10* and *HOXA9* promoter

Gene	Forward	Reverse
HOXA9 (Seq)	**F-** GGTTTTTGTTATAAAAATTATGATTGTAAA	**R-** AATTACCCAAAACCCCAATAATAAC
HOXA10 (Seq)	**F-** GTT GGG GTA GTT TTT ATA GTT TT	**R-** ATAACCCTTTCTAACTAACATTTCTTATAC
HOXA9 (CHIP)	**F-** ACGTAGTAGTTGCCCAGGGCC	**R-** TGCAGTTTCATAATTTCCGTGG
HOXA10 (CHIP)	**F-** CCC GAG CTG ATG AGC GAG TC	**R-** GCC AAA TTA TCC CAC AAC AAT GTC
MYOD1 (neg. Ctrl)	**F-** CCGCCTGAGCAAAGTAAATGA	**R-** GGCAACCGCTGGTTTGG
GAPDH (pos. Ctrl)	**F-** TACTAGCGGTTTTACGGGCG	**R-** TCGAACAGGAGGAGCAGAGAGCGA

Extended Experimental Procedures

DNA methylation profiling and methylation-specific clone sequencing

DNA was isolated from frozen tissue or cells using the AllPrep DNA/RNA Kit (Qiagen, 80204). A total of 600 ng of genomic DNA was treated with sodium bisulfite using the EZ DNA Methylation Kit (Zymo Research, D5001). After bisulfite treatment, purified DNA served as input for DNA methylation analysis on the Infinium HumanMethylation 450K BeadChip (Illumina) and for MS Clone Sequencing as previously described [1, 2]. PCR products for *HOXA9* (267 bp) and *HOXA10* (275 bp) encompassing 24 CpG sites (primers in Table S10) were cloned into a pCR2.1-TOPO vector according to the manufacturer's instructions (TOPO TA Cloning, Invitrogen). The plasmid was used to transform TOP10 competent cells. Ten to twenty cloned fragments per sample were sequenced using M13 primers (Sanger method, Microsynth CH-Balgach, Switzerland).

Selection of Illumina Infinium 450k probes

First, probes located in CGIs of HOX-signature genes – based on Illumina Infinium 450k annotation – were selected. This resulted in a list of 400 probes. In order to reduce the dimensionality of the DNA methylation data, we then performed a rational filtering of 450k probes, which were representative of the variability in DNA methylation observed for the first selection of probes. In a first step, Normed Principal component analysis (Normed PCA) was performed on all 400 probes. In a second step, we selected the 100 probes with the highest cumulative relative contributions of the decomposition of inertia [3] on the 3 first distinct axes. This strategy provided a set of the most variable probes after removing the unstructured variability (noise).

aCGH dataset data processing

The Lift Genome Annotations Tools was used to convert coordinates of 1986 BACs for the Assembly GRCh37 (https://genome.ucsc.edu/cgi-bin/hgLiftOver). After identification of BAC clones, Cy3/Cy5 ratio (Relative Ratio, RR) and average of signals per clone were computed. Data processing was completed by signal normalization to median RR per hybridization and computation of log2RR. An additional smoothing procedure was applied to remove wave bias for more accurate breakpoint detection in profiles as proposed by van de Wiel et al. [4]. CNA data was analysed by circular binary segmentation (CBS) [5, 6] performed on normalized *log2(RR)* values for each sample. The R packages DNAcopy and CGHcall [7] were used to performed CBS and to established copy number alteration events.

Description of selection of TCGA samples included in the analysis and data processing

To select samples with expression data with sufficient coverage of HOX signature genes we inspected annotation files of the two gene expression arrays used for TCGA samples, the Affymetrix U133A chip and a custom designed Agilent 244k chip. The Affymetrix U133A platform only covers 12 of the 21 HOX-signature genes, and therefore samples with only this gene expression data available were excluded from further analysis. The TCGA Agilent 244k chip has probes which measure expression levels of 19 HOX-signature genes. Applying both selection criteria to the TCGA GBM data resulted in the selection of 111 TCGA samples. The level 1 Agilent and Infinium data (raw) for these

samples were downloaded and processed as described below. Applying our stringent quality control criteria (see below) to both expression and DNA methylation data resulted in a remaining set of 106 TCGA samples (see Table S4 for list of IDs). Furthermore, level 3 SNP6 CNA data was available for 103 of the 106 selected samples, and was included for establishing the link between CNA and DNA methylation effects on gene expression. MicroRNA expression data were available for 96 of the 106 selected samples. Agilent level 2 expression data (probe-level normalized) were downloaded for 473 TCGA samples and were used for establishing population-wide HOX classification. Patient information and molecular subclass was taken from the annotation file available in Brennan et al. [[8] Supplemental Table 7]. For illustration of the analysis a data flowchart is presented in Figure S15.

Statistical method for the correlation between DNA methylation and expression

The two correlation matrices were directly compared by using a Monte-Carlo test based on RV-coefficient, a multivariate generalization of the squared Pearson correlation coefficient [9] without centering. After 9999 permutations, the simulated p-value was computed as suggested in [10]. The permutation test was performed by modified function from the R package ade4 [11, 12].

The stability of the mean correlation between DNA methylation and the HOX-signature was tested by Monte-Carlo rank sum tests for each CpG. The FDR method was used to correct the discrete p-values for multiple testing. However, the estimate (π_0) of the proportion of case for which the null hypothesis is true, was provided by bootstrap

procedure [13] to reduce the effect of the violating of continuity assumption. The implementation is available in the R package qvalue.

Analysis of chromosome 7-wide correlation between DNA methylation and gene expression

Probes measuring expression of RefSeq annotated genes were selected from the two different gene expression arrays (Affymetrix HU133 Plus 2, and TCGA Agilent custom design) based on the available annotation. If more than one probe was available per RefSeq gene, we selected the probe with the highest population-wide variability, based on the observed standard deviation (SD), to represent expression levels of the gene. This resulted in selecting 746 and 816 genes for the NCH_EORTC and TCGA datasets, respectively. Then the corresponding HM450k probes measuring DNA methylation in regions of the respective chromosome 7 RefSeq annotated genes were selected, and pair-wise correlations calculated (using Spearman's correlation coefficient). This resulted in vectors of multiple correlation coefficients per gene. For visualization and further analysis, we selected each gene-HM450k pair-wise correlation with the minimum coefficient, which most often resulted in finding the strongest anti-correlation. The correlation coefficients were plotted along the x-axis based on position of the genes on chromosome 7. In order to detect if the observed enrichment of anti-correlations at the HOXA locus was significantly different we performed gene set enrichment analysis [14] for the NCH_EORTC and TCGA data separately. For the NCH_EORTC data, 711 genes with negative correlation (of 746 on CHR7) were used as background against which the observed correlations of 10 HOXA genes were tested for enrichment. For the TCGA data

we tested for enrichment of 11 HOXA genes against the background of 791 chromosome 7 genes with negative correlations (of 846).

Copy number status of CHR7 in NCH_EORTC samples

CHR 7 status for EORTC samples was estimated by fitting a two normal mixture model to the segmented copy number obtained from the BAC CGH data using the R package CGHcall [7].

Copy number status of CHR7 in TCGA samples

A two normal mixture model was fitted to the weighted means of the segmented copy number (TCGA Agilent SNP6 Level 3) using EM-algorithm, as implemented in the R package “mixtools” [15]. In order to obtain stable estimations, priors equal to 0.3 (normal CHR7) and 0.7 (gain of CHR7) were set. This limits the potential effects of local minima.

Pair-wise correlation between *HOXA10* DNA methylation and gene expression

Spearman correlation coefficients were calculated for probes measuring expression levels of HOXA10 and 28 Infinium 450k probes annotated to be located in two *HOXA10*—associated CGIs. The Affymetrix U133 Plus 2 platform contains several probes annotated to measure *HOXA10* expression levels. One representative probe (214651_s_at) was selected based on highest observed variability (SD) in the population of 59 samples. NCH_EORTC samples were then separated according to CHR 7 status (normal or gain), and correlation coefficients and test statistics were calculated for both sub-population separately. For the TCGA data, Agilent probe A_24_P77904 measures expression levels

of *HOXA10*. Spearman correlation coefficients were calculated between this probe and the same 28 Infinium 450k probes as above. TCGA samples were also stratified by CHR7 status.

Pair-wise correlation between selected HOX signature DNA methylation probes and mean signature expression levels.

Analog to the method described above, the correlation coefficients between DNA methylation and the mean expression levels of the HOX signature in both data sets were calculated. Both NCH_EORTC and TCGA samples were again subdivided according to their CHR7 status.

RNA-Seq of glioma sphere transcriptomes and data analysis

Total RNA was isolated from biological duplicates of four glioma sphere lines (LN-2207GS, LN-2540GS, LN-2669GS, LN-2683GS) using the AllPrep DNA/RNA Kit (Qiagen, 80204). Directional total RNA libraries were prepared from rRNA-depleted total RNA using TruSeq Stranded Total RNA with Ribo-Zero Gold (Epicentre, Illumina), followed by paired-end sequencing on Illumina Hiseq (PE 2x50 bp; NXTGNT, University of Gent, Belgium). We performed splice-aware alignment of RNA-Seq reads using tophat2 to the target genome (Ensembl assembly GRCH37.73, hg19) and visualized the resulting bp-level read counts (normalized by library size, resulting in **R**eads-**P**er-**M**illion) using R/Bioconductor. Aligned reads of each library were assembled using cufflinks2 with the Ensembl annotation GRCh37.73 as a reference. Parameters for cufflinks2 were chosen so that novel isoforms & transcripts would be reported. The

transcriptomes assembled from each library were then merged using the cuffmerge/cuffcompare programs of the cufflinks2 suite. The resulting consensus transcriptome was visualized using Integrative Genome Viewer [16] and inspected for the presence of novel transcripts and isoforms at the *HOXA* locus. RNA-Seq expression data are available upon request (privacy issues).

Chromatin Immunoprecipitation followed by quantitative PCR (ChIP-qPCR)

Chromatin was prepared using the MAGnify Chromatin Immunoprecipitation System (Invitrogen) according the manufacturer's protocols as previously described [2]. For the immuno-precipiation equivalent amounts of, either anti-Histone-H3 (Positive control, Abcam, AB1791), anti-trimethyl-Histone H3 (Lys 4) (C42D8; #9751, Cell Signaling Technology; 09/2009), anti-trimethyl-Histone H3 (Lys 27) (C36B11; 9733, Cell Signaling Technology), anti-trimethyl H3 (Lys 36) (D5A7; #4909s, Cell Signaling Technology 11/2009), and normal rabbit IgG (negative control, Invitrogen) were added and incubated according to the protocol. Purified DNA was quantified by quantitative real-time PCR. Primers for *HOXA10 and HOXA9*, and the two controls, *GAPDH* as representative of a gene with an open chromatin state (EZ ChIP. Chromatin Immunoprecipitation Kit) and *MYOD1* as representative of a gene with a closed chromatin state [17] are listed in Table S10. The experiments were performed in biological duplicates.

Supplemental References

1. Bady P, Sciuscio D, Diserens AC, Bloch J, van den Bent MJ, Marosi C, Dietrich PY, Weller M, Mariani L, Heppner FL, et al: **MGMT methylation analysis of glioblastoma on the Infinium methylation BeadChip identifies two distinct CpG regions associated with gene silencing and outcome, yielding a prediction model for comparisons across datasets, tumor grades, and CIMP-status.** *Acta Neuropathol* 2012, **124:**547-560.
2. Sciuscio D, Diserens AC, van Dommelen K, Martinet D, Jones G, Janzer RC, Pollo C, Hamou MF, Kaina B, Stupp R, et al: **Extent and patterns of MGMT promoter methylation in glioblastoma- and respective glioblastoma-derived spheres.** *Clin Cancer Res* 2011, **17:**255-266.
3. Lebart L, Morineau A, Piron M: *Statistique exploratoire multidimensionnelle (3e édition).* Paris: Dunod; 2000.
4. van de Wiel MA, Brosens R, Eilers PH, Kumps C, Meijer GA, Menten B, Sistermans E, Speleman F, Timmerman ME, Ylstra B: **Smoothing waves in array CGH tumor profiles.** *Bioinformatics* 2009, **25:**1099-1104.
5. Olshen AB, Venkatraman ES, Lucito R, Wigler M: **Circular binary segmentation for the analysis of array-based DNA copy number data.** *Biostatistics* 2004, **5:**557-572.
6. Venkatraman ES, Olshen AB: **A faster circular binary segmentation algorithm for the analysis of array CGH data.** *Bioinformatics* 2007, **23:**657-663.

7. van de Wiel MA, Kim KI, Vosse SJ, van Wieringen WN, Wilting SM, Ylstra B: **CGHcall: calling aberrations for array CGH tumor profiles.** *Bioinformatics* 2007, **23:**892-894.

8. Brennan CW, Verhaak RG, McKenna A, Campos B, Noushmehr H, Salama SR, Zheng S, Chakravarty D, Sanborn JZ, Berman SH, et al: **The somatic genomic landscape of glioblastoma.** *Cell* 2013, **155:**462-477.

9. Heo M, Gabriel KR: **A permutation test of association between configurations by means of the RV coefficient.** *Communications in Statistics - Simulation and Computation* 1997, **27:**843-856.

10. Davison AC, Hinkley DV: *Bootstrap methods and their application.* Cambridge University Press; 1997.

11. Chessel D, Dufour AB, Thioulouse J: **The ade4 package-I- One-table methods.** *R News* 2004, **4:**5-10.

12. Dray S, Dufour AB, Chessel D: **The ade4 package-II: Two-table and K-table methods.** *R News* 2007, **7:**47-52.

13. Storey JD, Taylor JE, Siegmund D: **Strong control, conservative point estimation and simultaneous conservative consistency of false discovery rates: a unified approach.** *J R Statist Soc B* 2004, **66:**187-205.

14. Subramanian A, Tamayo P, Mootha VK, Mukherjee S, Ebert BL, Gillette MA, Paulovich A, Pomeroy SL, Golub TR, Lander ES, Mesirov JP: **Gene set enrichment analysis: a knowledge-based approach for interpreting genome-wide expression profiles.** *Proc Natl Acad Sci U S A* 2005, **102:**15545-15550.

15. Benaglia T, Chauveau D, Hunter DR, Young DS: **mixtools: An R Package for Analyzing Mixture Models.** *Journal of Statistical Software* 2009, **32:**??-??
16. Thorvaldsdottir H, Robinson JT, Mesirov JP: **Integrative Genomics Viewer (IGV): high-performance genomics data visualization and exploration.** *Brief Bioinform* 2013, **14:**178-192.
17. Taniguchi H, Yamamoto H, Akutsu N, Nosho K, Adachi Y, Imai K, Shinomura Y: **Transcriptional silencing of hedgehog-interacting protein by CpG hypermethylation and chromatic structure in human gastrointestinal cancer.** *J Pathol* 2007, **213:**131-139.
